# Finding a path: local search behavior of *Drosophila* larvae

**DOI:** 10.1242/jeb.249913

**Published:** 2025-11-13

**Authors:** Jessica Kromp, Tilman Triphan, Andreas S. Thum

**Affiliations:** ^1^ Department of Genetics, Leipzig University, 04103 Leipzig, Germany; ^2^ German Centre for Integrative Biodiversity Research (iDiv) Halle-Jena-Leipzig, Leipzig, Germany

**Keywords:** *Drosophila* larvae, Behavior, Orientation, Chemosensation, Olfaction

## Abstract

Orientation and navigation are essential features of animals living in changing environments. Typically, animals integrate a variety of allothetic and idiothetic cues to achieve their navigational goals. Allothetic cues, such as visual or chemical landmarks from the environment, provide an external frame of reference. In contrast, idiothetic cues are based on internal proprioceptive feedback and internal copies of motor commands.

When *Drosophila* larvae are exposed briefly to a Teflon container holding a food stimulus, they show a characteristic behavior as soon as the container is removed: they briefly crawl away from the detected resource, remain in its vicinity and then return to the area where they experienced the earlier stimulus. We quantified this behavior with respect to the chemosensory nature of the stimulus, starvation time and genetic background of the larvae. We conclude that this behavior represents a centered local search. Furthermore, we exclude various external stimuli (vision and taste), which suggests that possibly idiothetic as opposed to allothetic cues have a stronger influence on the larval local search behavior. In the long term, this behavioral description will enable us to gain insights into the comparability of larval foraging strategies. We also want to investigate whether, despite the simpler organization of the larval brain and the alleged lack of a central complex, a brain region that is important for orientation and navigation in adult *Drosophila* and other insects, there are common solutions for the brain circuits underlying search behavior.

## Teaser

**Summary:** *Drosophila* larvae show a local search behavior after a brief food exposure, suggesting internal navigation mechanisms despite the lack of complex brain structures in adult insects.

## INTRODUCTION

Navigation and orientation in the environment are indispensable for an animal's survival. Orientation refers to an animal's ability to use environmental cues to determine and maintain its position or heading relative to the surrounding environment. In contrast, navigation is a goal-directed process that involves the integration of spatial information to estimate both the animal's own position and that of a specific target, enabling directed movement toward that goal (Gould and Gould, 2012; Mast, 1941). To reach their navigational goals, such as food sources and mating partners, or to avoid specific places, animals rely on allothetic (from the environment) and idiothetic cues (from its own movements) (Bell, 1990; Bures et al., 1998; Mittelstaedt and Mittelstaedt, 2001). Animals often follow visual or olfactory stimuli to find their targets (Zeil, 2023; Zjacic and Scholz, 2022). Bees, living in feature-rich habitats, absolve orientation flights to get familiar with the surrounding environmental features before searching for distant food (Degen et al., 2016; Menzel et al., 1996). In featureless habitats, animals can utilize self-motion cues and internal representations to navigate, a process called internal path integration (Muller and Wehner, 1988). Desert ants, for example, live in habitats with few environmental cues; they return home in straight lines after traveling long, winding paths when leaving the nest. This navigation ability is facilitated by the integration of various sensory cues, including celestial and polarization compass cues, along with a pedometer (Muller and Wehner, 1988; Wittlinger et al., 2006).

The fruit fly *Drosophila melanogaster* is a widely used model organism to characterize the neuronal and molecular mechanisms of a multitude of natural behaviors such as locomotion, chemosensory discrimination, courtship, learning and memory, as well as foraging (Cognigni et al., 2018; Gomez-Marin et al., 2010; Heisenberg, 2003; Mahishi and Huetteroth, 2019; Rings and Goodwin, 2019; Vosshall and Stocker, 2007; Widmann et al., 2018). After consuming a food droplet, adult *Drosophila* perform a specific sequence of repetitive locomotory patterns starting with a pause, a walk away from the stimulus, a reversal and a return to the previously encountered food source (Corfas et al., 2019; Kim and Dickinson, 2017; Murata et al., 2017; Titova et al., 2023). A similar locomotory pattern was observed in *Drosophila* larvae tested in patchy environments. Depending on the food quality, larvae decreased their speed, performed higher number of pauses and turned to the center of the patch when reaching its border (Wosniack et al., 2022). Whether larvae perform an adult-like local search after the removal of the food stimulus is not known and was therefore investigated here.

The locomotion of *Drosophila* larvae consists of an alternating sequence of runs and oriented turns (Berni et al., 2012; Gomez-Marin et al., 2011). The modulation of locomotion is usually based on allothetic cues such as odors or tastes, which are perceived via the peripheral and pharyngeal nervous system. Olfactory information is received via specific olfactory receptors (ORs) located in 21 olfactory receptor neurons (ORNs) (per body half) within the dorsal organ (Fishilevich et al., 2005; Larsson et al., 2004; Singh and Singh, 1984). In contrast, taste information activates a group of the approximately 55 gustatory receptor neurons (GRNs) (per body half) organized in four external (terminal, ventral, dorsal and labial organ) and four internal head organs (dorsal, ventral, posterior pharyngeal sensilla, dorsal pharyngeal organ) (Gendre et al., 2004; Python and Stocker, 2002; Richter et al., 2024; Rist and Thum, 2017). When a larva approaches a food source, it modulates its locomotion by making fewer turns, thereby moving in a straighter line. To initiate a turn, the larva scans the local odor gradient by moving its head from side to side, a behavior known as head casting. The larva then performs its next run most often towards the odor source (Gershow et al., 2012; Gomez-Marin et al., 2011).

In the brains of adult flies and many other insects, the central complex (CX) allows the animal to modulate its turn rate and turn direction (Basu and Nagel, 2024; Honkanen et al., 2019). In general, the CX consists of four compartments: the ellipsoid body (EB), the fan-shaped body, the protocerebral bridge (PB) and the paired noduli (Hanesch et al., 1989). Recently, the asymmetric body was proposed to be a fifth compartment (Wolff and Rubin, 2018). Besides its involvement in the orientation toward stable landmarks or the sun, the CX is known to mediate path integration based on internal representations by recalculating the animal's recent spatial position via self-motion cues (Giraldo et al., 2018; Green et al., 2017, 2019; Seelig and Jayaraman, 2015). The current position is established as an activity ‘bump’ within the EB owing to the global inhibition of EB/PB gall neurons and PB/EB noduli neurons and updated by reciprocal exciting interaction of these neurons (Green et al., 2017; Vafidis et al., 2022). However, in larvae, the corresponding neurons are largely undifferentiated, and the few existing parts are non-functional (Farnworth et al., 2020; Riebli et al., 2013). In contrast, it has been shown that the mushroom body (MB), which serves as the integration and memory center, can modulate larval locomotion. Activation of specific MB output neurons prompts the larva to halt and begin head casting, whereas other neurons induce the opposite behavior, causing larvae to suppress turns and move in a straight path (Eichler et al., 2017; Eschbach et al., 2021; Pauls et al., 2010; Saumweber et al., 2018).

To better understand the orientation and foraging behavior of larvae, we have developed a novel behavioral assay. Our findings suggest that upon detecting a chemosensory stimulus, larvae initiate a local search behavior, which continues for several minutes after the stimulus has been removed. Olfactory and gustatory stimuli, such as apple juice and yeast, trigger local search behavior. Notably, the larval feeding state and the agarose substrate used in the test arena do not significantly influence this behavior. In the long term, the new behavioral paradigm will enable us to identify the neuronal, molecular, and physiological foundations of larval foraging behavior.

## MATERIALS AND METHODS

### Fly strains

Flies were reared on standard food medium at 25°C, at a relative humidity of 60–80% and a 14 h:10 h light:dark cycle. Larvae with the *white* mutation (*w^1118^*, Bloomington Stock Center no. 3605) were used to determine the parameters for the standard experiment. To test whether the *white* mutation impacts local search behavior, we compared them with wild type Canton-S (*WT-CS*, Bloomington Stock Center no. 9515) larvae.

### Recording settings

Larval tracking was performed under red light via the GigE Basler acA1300–60gm NIR camera (version: 106202-22) using a FTIR-based imaging method (FIM) table (Risse et al., 2013) and an adapted recording program that was established in an earlier study in the lab (Schumann and Triphan, 2020). Image sequences were recorded with two frames s^−1^. Infrared light emitting diodes (IR-LEDs) were used for illumination. For an optimal recording of larvae, the recording settings were established to a resolution of 1024×1024, an exposure rate of 2000, a gamma of 3.99 and a gain of 1.0. The FIM LED level was set to 6.

### Preparation of larvae

A spatula tip of food containing 6- to 7-day-old larvae was transferred into a Petri dish lid and washed with water. Subsequently, the cleaned animals were collected in a drop of water within a second Petri dish lid. Dependent on the specific starvation time, larvae remained in this drop of water (for 1 or 3 h hours of starvation) or were used directly (starvation time of 0 h). Owing to the gradual evaporation of water, larvae scheduled to undergo a 6 h of starvation were transferred to a vial containing water.

### Preparation of chemosensory stimuli

Food odors, serving as chemical stimuli, were freshly prepared each day up to 1 h before the start of the initial trial of an experiment and stored in Eppendorf tubes. Five to ten min before the experiment started, containers (Teflon, custom-made) were filled with 10 μl of the respective stimuli. Specifically, the following procedures were implemented for each individual stimulus. (1) Apple juice: containers were freshly prepared before each trial started. To test the effect of various apple juice concentrations (25%, 50%, 75% and 100%), 100% apple juice (standard supermarket quality) was diluted with the respective amount of water. (2) Odor preparation: amyl acetate (AM) (Fluka cat. no. 46022; CAS no. 628-63-7) was diluted with paraffin oil (Fluka cat. no. 76235, CAS no. 8012-95-1) to adjust a concentration of 1:250, 1:1000 and 1:10,000. The odors benzaldehyde (BA) (Fluka cat. no. 12010, CAS no.100-52-7) and 3-octanol (3-OCT) (Fluka cat. no. W358118, CAS no. 589-98-0) were used undiluted. The prepared containers were used for up to three consecutive trials. (3) Yeast: a cube of fresh baker’s yeast was weighed and diluted with the respective amount of water to reach the tested yeast concentrations (25%, 50%, 75% and 100%, e.g. for 25%: 0.25 g yeast+0.75 g water). Note that due to the high viscosity of the 75% yeast solution, the containers were filled with a pipette tip of this solution. For 100% yeast, a small piece of yeast was placed into the container. Containers were freshly prepared before starting a new trial. Please note that we experienced slight differences in the attractiveness of the yeast container, probably owing to variations of the yeast quality and yeast strain obtained from supermarkets. Therefore, we recommend the use of fermented yeast, which did not induce differences in attractiveness. (4) Fermented yeast: 0.05 g sucrose was added per gram yeast solution (25%, lukewarm water) and stirred. The solution was prepared approximately 1 h prior to the initiation of the first trial and stored in a vial sealed with a ceaprene stopper. For the additional yeast-dependent gustatory stimulus, the lid of the container was moistened with the fermented yeast solution. Containers were replaced with new ones before a new trial started. After a 4 h period, the solution was replaced.

### Preparation of the tracking area

**Fig. 1.**
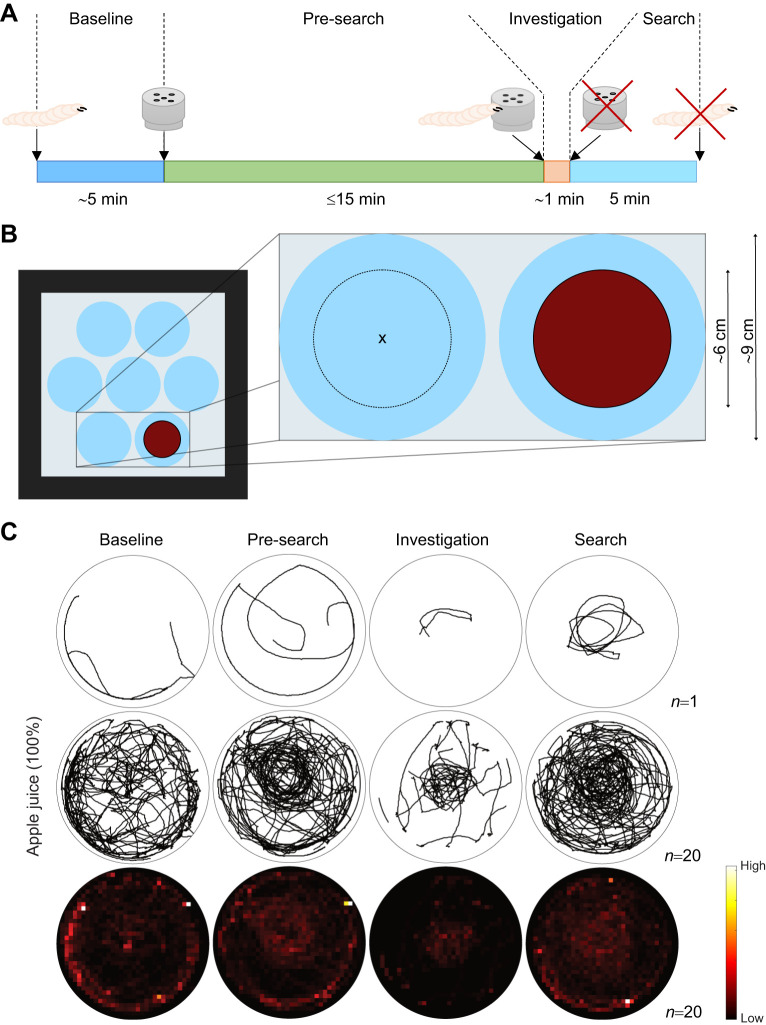
***Drosophila melanogaster* larvae perform a local search.** (A) The local search paradigm. Individuals were placed into the center of a search arena and had 5 min to explore it (baseline phase). Next, an odor-filled container was placed into the arena center and initiated the pre-search phase. If the larva interacted with the container (physical contact with its head) within 15 min, a 1 min investigation phase started. This was terminated by removing the container. Subsequently, the larvae were observed for 5 min (search phase). Larvae that failed to interact with the container were excluded. (B) Paradigm arrangement. At the top of the acrylic plate up to seven agarose patches were placed. Centralized at each agarose patch, a lid (red circle) is arranged and defines the larval search area (*d*=6 cm, *h*=0.8 cm). Over the course of the experiment, larvae and container were placed in the center of this area (marked with X). (C) Walking trajectories/residency plots with apple juice (100%). Columns show the crawling path and residency during the four test phases (baseline, pre-search, investigation and search) row-wise for a single larva or the entire test group. Larvae were tested after 1 h of starvation.

**Fig. 2.**
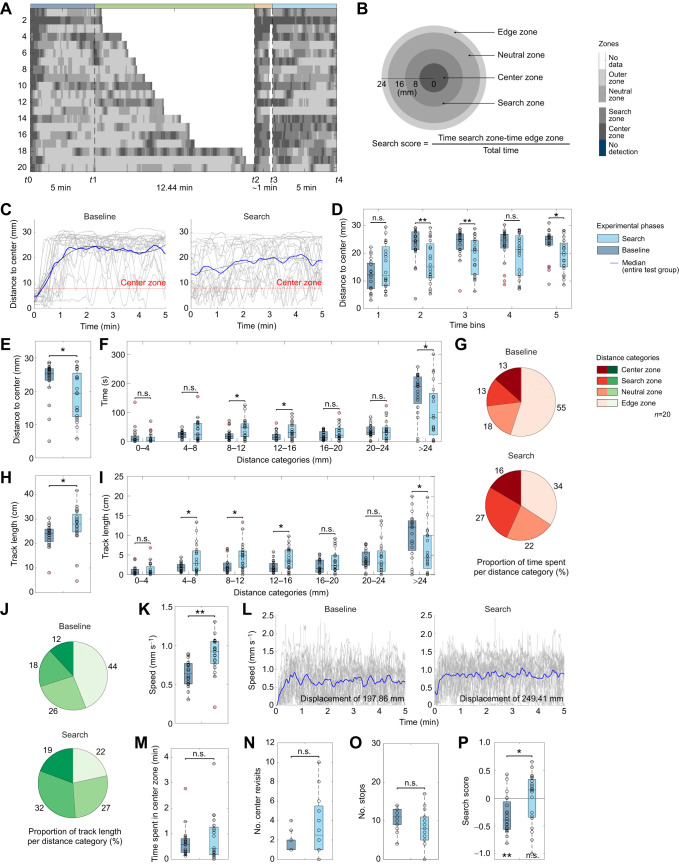
**Evaluation of tracking data of larvae exposed to apple juice (100%).** (A) Grayscale position plots. Shown is the individual larval spatio-temporal distribution in the arena separated for the baseline, pre-search, investigation and search phase (indicated at the top). The larvae are sorted top-down by their individual time needed to find the container. The shade of gray represents the larva's position, transitioning from dark to light gray as it moves from the center zone through the search and neutral zones to the edge zone. (B) Overview of the arena's zonal subdivision into center (0–8 mm), search (8–16 mm), neutral (16–24 mm) and edge zone (<24 mm, increasing brightness). The formula for calculating the search score is shown below. (C) Distance to center – progression over time. Displayed are the means (blue line) and the individual (gray lines) distances to center. Initially, the larvae rapidly left the arena center and remained near the edge (baseline) while they stayed more centralized during the search phase. (D) Distance to center in 1 min time intervals. While larvae showed no distance differences between baseline and search phase in the first (*P*=0.135) and fourth (*P*=0.126) minute, they remained closer to the center during the search phase during the second (*P*=0.004), third (*P*=0.003) and fifth (*P*=0.037) minute. (E) Distance to center box plot. Compared are the median larval distances during the baseline and search phase. Larvae reduced their distances to center during the search phase significantly compared with the baseline (*P*=0.019). (F) Time spent per distance category compared for baseline and search phase. After the exposure to apple juice larvae spent more time at a distance of 8–16 mm (8–12 mm: *P*=0.038, 12–16 mm: *P*=0.018) and less time in the outermost distance category (>24 mm: *P*=0.014). The time spent within the other distances was unaffected (0–4 mm: *P*=0.446; 4–8 mm: *P*=0.167; 16–20 mm: *P*=0.117; 20–24 mm: *P*=0.809). (G) Proportion of time spent per distance category. Pie charts represent the proportion of time spent per distance category counterclockwise from center (dark red) to edge (beige). (H) Track length. The plots display the total track length covered during the respective phases. Larvae crawled longer paths after the interaction with the container (*P*=0.017). (I) Track length per distance category. Shown is the track length covered per distance category compared with the respective phases. After exposure to apple juice, larvae crawled longer paths in a distance of 4–16 mm (4–8 mm: *P*=0.040; 8–12 mm: *P*=0.023; 12–16 mm: *P*=0.015) while lowering the crawled distance in the outermost category (>24 mm: *P*=0.010). The track length within the remaining categories was unaffected (0–4 mm: *P*=0.396; 16–20 mm: *P*=0.145; 20–24 mm: *P*=0.841). (J) Proportion of track length crawled per distance category. Pie charts represent the respective proportion counterclockwise from center (darkest green) to edge (lightest green). (K) Speed box plot comparing the larval speed during the respective phases. After the container interaction, larvae increased their speed (*P*=0.001, median_base|search_=0.64 mm s^−1^|0.92 mm s^−1^). (L) Speed progression over time. The graphs display the test group's mean (blue line) and individual speed (gray lines) over time, both averaged over ten frames. (M) Time spent in center zone. The comparison of baseline and search phase revealed no differences in the time spent within the center zone (*P*=0.380). (N) Number of center revisits. The box plot visualizes the number of center revisits (crossing of the center zone boundary, inward direction) during the respective phases. The number of revisits was unaffected by the container interaction (*P*=0.092). (O) Number of stops. Compared are the number of larval stops during baseline and search phase. There is no significant change in the number of stops (*P*=0.058). (P) Search score displaying the larval preference toward the search (positive values) or the edge (negative values) zone. The results show a larval baseline preference for the edge (*P*=0.003) while they behave neutral during the search phase (*P*=0.852). The initial center avoidance was significantly reduced after odor presentation (*P*=0.014). The larvae were tested after 1 h starvation. For the statistical evaluation the one-sample and two-sample Wilcoxon signed-rank test were performed. *P≤0.05, **P≤0.01.

**Fig. 3.**
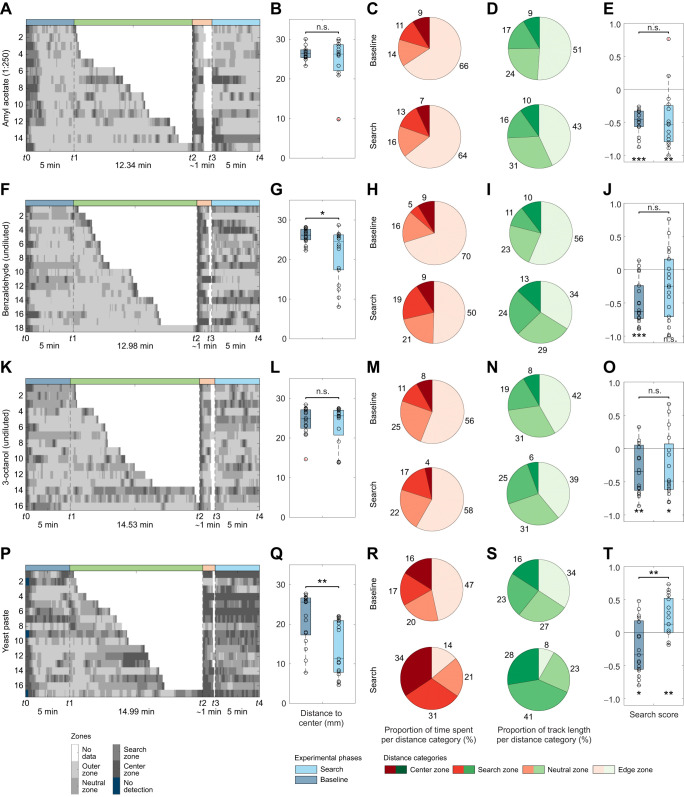
**Impact of different chemical stimuli on the local search.** Larvae were tested in the local search paradigm to investigate the effect of different chemical stimuli: (A–E) amyl acetate (*n*=15), (F–J) benzaldehyde (*n*=18), (K–O) 3-octanol (*n*=16) and (P–T) yeast (*n*=17). (A,F,K,P) Grayscale position plots for each experiment. (B,G,L,Q) Distance to center box plot. Larvae exposed to benzaldehyde (G: *P*=0.018) and yeast (Q: *P*=0.002) have a reduced distance to center after container removal while the presentation of AM and 3-Oct have no effect (B: *P*=0.934, G: *P*=0.756). (C,H,M,R) Proportion of time spent per distance category. Pie charts represent the respective proportion counterclockwise from center (dark red) to edge (beige). (D,I,N,S) Proportion of track length crawled per distance category. Pie charts represent the respective proportion counterclockwise from center (darkest green) to edge (lightest green). (E,J,O,T) Search score displaying the larval preference toward the search (positive values) or the edge (negative values) zone. The results show that each test group avoids the center during the baseline phase (E: *P*<0.001; J: *P*<0.001; O: *P*=0.010; T: *P*=0.031). Only larvae exposed to yeast preferred the search zone during the search phase (E: *P*=0.005; J: *P*=0.078; O: *P*=0.049; T: *P*=0.007) and increased their search score significantly (E: *P*=0.793, J: *P*=0.145, O: *P*=0.796, T: *P*=0.001). The larvae were tested after a starvation time of 1 h. For the statistical evaluation the one-sample and two-sample Wilcoxon signed-rank test were performed. *P≤0.05, **P≤0.01, ***P<0.001.

**Fig. 4.**
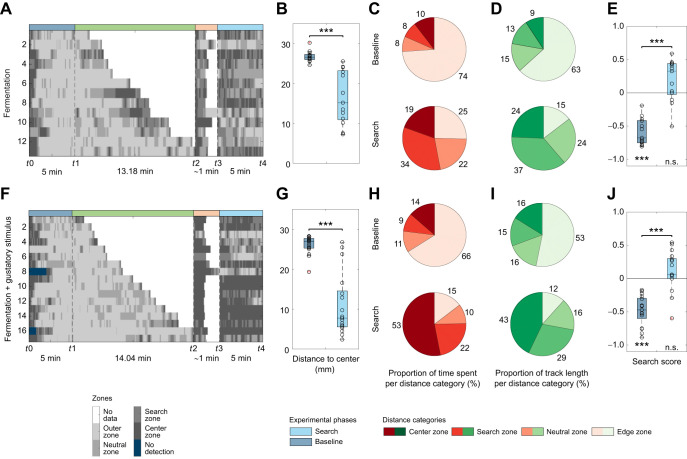
**Impact of fermented yeast on the local search behavior.** Larvae were exposed to either (A–E) fermented yeast (only olfactory stimulation, *n*=13) or (F–J) fermented yeast with a yeast moistened lid (combination of olfactory and gustatory stimulation, *n*=17). (A,F) Grayscale position plots for each experiment. (B,G) Distance to center box plot. The presentation of each yeast stimulus significantly decreased the larval distance to center (B: *P*<0.001; G: *P*<0.001). (C,H) Proportion of time spent per distance category. Pie charts represent the respective proportion counterclockwise from the center (dark red) to the edge (beige). (D,I) Proportion of track length crawled per distance category. Pie charts represent the respective proportion counterclockwise from the center (darkest green) to the edge (lightest green). (E,J) Search score. The search score displays the larval preference toward the search (positive values) or the edge (negative values) zone. The results show that each test group avoids the center during the baseline phase (E: P<0.001; J: P<0.001) but display a neutral behavior during the search phase (E: *P*=0.080; J: *P*=0.119) by increasing their search score significantly (E: *P*<0.001, J: *P*<0.001). The larvae were tested after a starvation time of 1 h. For the statistical evaluation, one-sample and two-sample Wilcoxon signed-rank tests were performed. ***P<0.001.

**Fig. 5.**
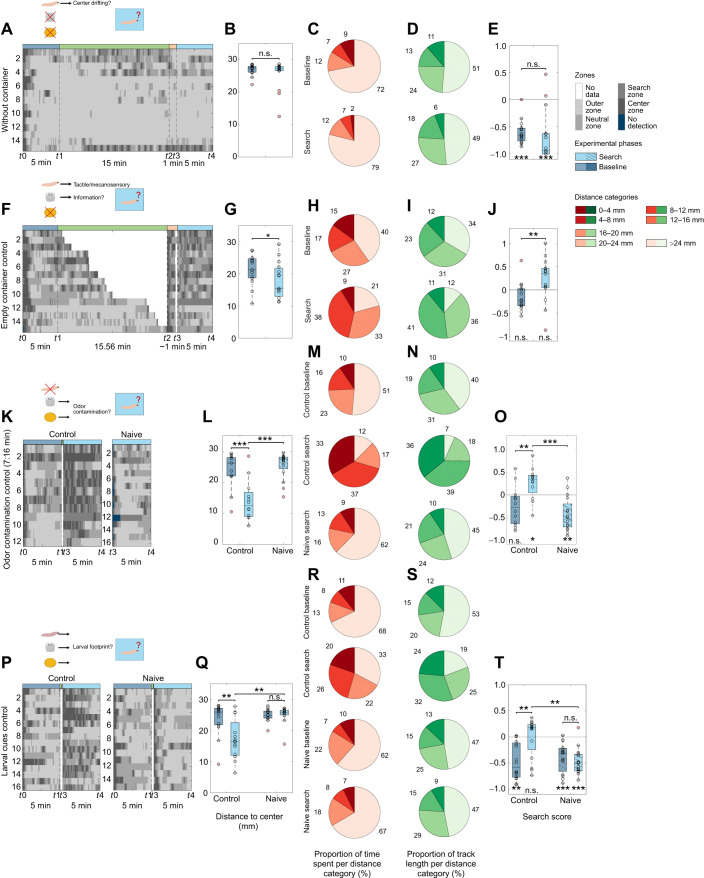
**Neither temporal effects nor potential lingering chemical cues affect the local search.** Larvae were tested via the local search paradigm to investigate the effect of the (A–E) time (without container, *n*=15), (F–J) an empty container (*n*=15), (K–O) potentially remaining odor cues (odor contamination control, *n*=12/16) or (P–T) cues provided by the larvae (larval cues control, *n*=16/15). (A,F,K,P) Grayscale plots for each experiment. (B,G,L,Q) Distance to center box plots. The interaction with an empty container led to a reduced distance to center (G: *P*=0.022) while larvae did not reduce their distance to center as a function of time (B: *P*=0.340). The tests with naïve animals showed that behavioral controls reduced their distance to center after the container interaction while naïve animals on odor or larval exposed test plates showed a baseline-like behavior (G: *P*_control_<0.001, *P*_naïve-base_=0.593, *P*_naïve-search_<0.001; Q: *P*_control_=0.008, *P*_naive_=0.359). (C,H,M,R) Proportion of time spent per distance category. Pie charts represent the respective proportion counterclockwise from center (dark red) to edge (beige). (D,I,N,S) Proportion of track length crawled per distance category. Pie charts represent the respective proportion counterclockwise from center (darkest green) to edge (lightest green). (E,J,O,T) Search score. Without a container as stimulus, larvae avoided the center during the baseline and search phase (E: *P*_base_<0.001, *P*_search_<0.001) and therefore did not change their search score (*P*=0.309). After interaction with an empty container, larvae significantly increased their search score (J: *P*=0.005) even though there was no additional stimulus (*P*_base|search_=0.064|0.073). Naïve larvae (i.e. first put into the arena in the search phase) avoided the search zone during the test (O: *P*_naïve_=0.043) while the behavioral control behaved neutrally during the baseline phase and preferred the center after the container interaction (*P*_base_=0.064, *P*_search_=0.043). When replacing larvae after the investigation phase with naïve animals, the naïve animals' search score was significantly different from behavioral control animals (T: *P*_naïve-base_=0.330, *P*_naïve-search_<0.001, *P*_base-search_=0.008). Larvae were tested after 1 h starvation. Naïve larvae were placed after a stimulus propagation time of 7:16 min (mean time the larvae needed to find the container during fermentation experiments). The behavioral controls and naïve larvae were tested by using fermented yeast. For the statistical evaluation the one-sample and two-sample Wilcoxon signed-rank test were performed. For the comparison of naïve larvae with the behavioral control the Mann–Whitney *U*-test was used. *P≤0.05, **P≤0.01, ***P<0.001.

**Fig. 6.**
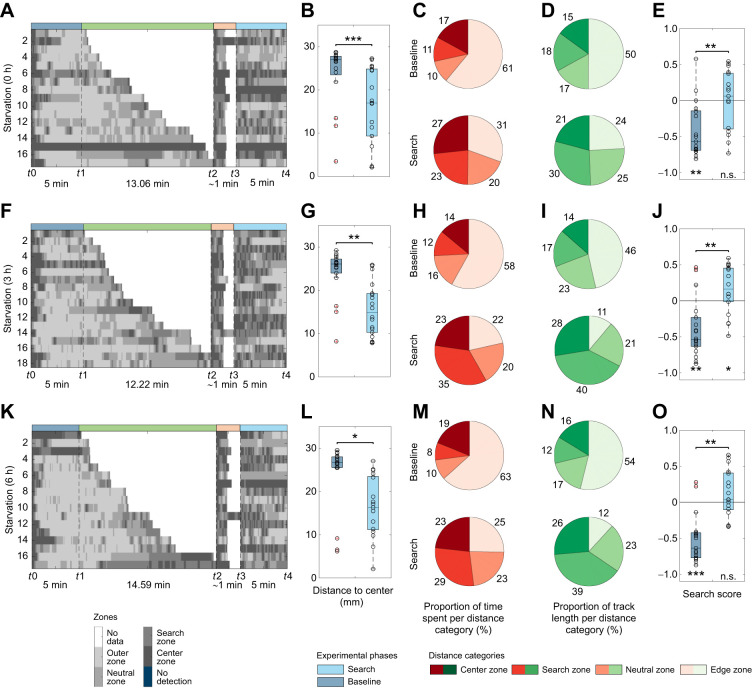
**Effect of starvation time on the local search.** Larvae were tested via the local search paradigm to investigate the effect of starvation time of (A–F) 0 h (*n*=17), (F–J) 3 h (*n*=18) or (K–O) 6 h (*n*=17) on the local search. (A,F,K) Grayscale plots for each experiment. (B,G,L) Distance to center box plots. All test groups reduced their distance to center significantly after the container interaction (B: *P*<0.001; G: *P*=0.006; L: *P*=0.019). (C,H,M) Proportion of time spent per distance category. Pie charts represent the respective proportion counterclockwise from center (dark red) to edge (beige). (D,I,N) Proportion of track length crawled per distance category. Pie charts represent the respective proportion counterclockwise from center (darkest green) to edge (lightest green). (E,J,O) Search score. All test groups preferred the edge before the odor container was presented and preferred the search zone during the search phase after a starvation time of three hours only (E: *P*_base_=0.003, *P*_search_=0.847; J: *P*_base_=0.003, *P*_search_=0.039; O: *P*_base_<0.001, *P*_search_=0.196). However, increasing search scores were observed for all the three test groups (E: *P*=0.001, J: *P*=0.003, O: *P*=0.002). The larvae were tested using fermented yeast as stimulus. For the statistical evaluation, one-sample and two-sample Wilcoxon signed-rank tests were performed. *P≤0.05, **P≤0.01, ***P<0.001.

**Fig. 7.**
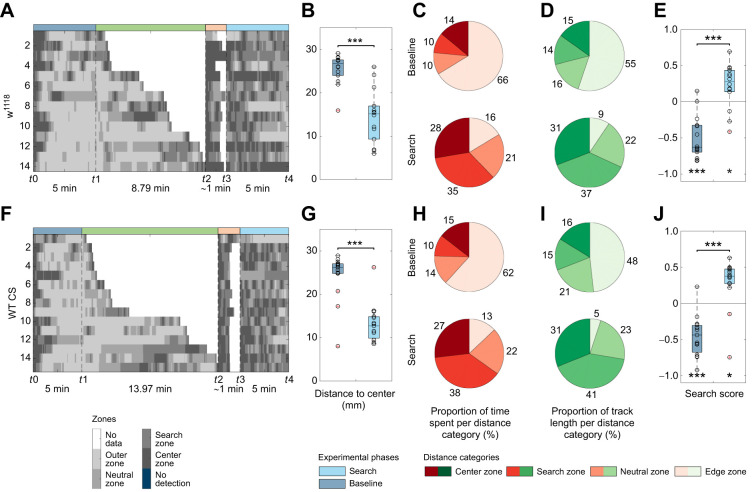
**Influence of the genetic background on the local search.** To test whether the genetic background of (A–F) *w^1118^* (*n*=14) impacts the search behavior we compared them with (F–J) *WT-CS* (*n*=15). (A,F) Grayscale position plots for each experiment. (B,G) Distance to center box plots. In comparison to the baseline phase, both test groups remained closer to the center after the container was removed (B: *P*<0.001; G: *P*<0.001). (C,H) Proportion of time spent per distance category. Pie charts represent the respective proportion counterclockwise from center (dark red) to edge (beige). (D,I) Proportion of track length moved per distance category. Pie charts represent the respective proportion counterclockwise from center (darkest green) to edge (lightest green). (E,J) Search score. Both test groups avoided the center during the baseline phase (E: *P*<0.001; J: *P*<0.001). After the container was removed, both groups preferred the search zone (E: *P*=0.025; J: *P*=0.010) and, therefore, increased their search score significantly (E: *P*<0.001; J: *P*<0.001). Larvae were tested after a starvation time of 1 h using fermented yeast as stimulus. For the statistical evaluation, one-sample and two-sample Wilcoxon signed-rank tests were performed. *P≤0.05, ***P<0.001.

Agarose plates (85 mm diameter, cat. no. 82.1472, Sarstedt, Nümbrecht) containing the specified agarose concentrations (0.8%, 1.4% and 2.0%) were freshly prepared daily at least 30 min before the start of the initial trial. The following concentrations were used in the respective figures: 0.8% in Figs 1, 2, 3A–E,P–T; 1.4% in Figs 3F–O, 5K–T and 7; 2.0% in Figs 4, 5A–J and 6. Agarose layers had a maximum thickness of 2 mm as established by Risse et al. (2013). The size of the FIM table enables us to conduct up to seven experiments simultaneously. Agarose patches with a diameter of 8.5 cm were positioned at the FIM table. At the beginning of the experiment, a larva was positioned at the center of an arena, and during the course of the experiment, a container was subsequently placed at the same central position (marked with an X in Fig. 1). Each arena was covered with an opaque lid (*d*=6 cm, *h*=0.8 cm) which reduces the background noise during tracking, defines the arena's boundaries and prevents the larva from leaving the area. Larval behavior can be continuously monitored in real time using the video tracking system's display. The inside walls of these lids were roughened with a file to reduce the likelihood of larvae climbing up the lid. A new agarose plate was used for each experiment.

### Local search paradigm

Experiments started with the transfer of a single larva via a brush to the arena center. With this transfer a 5 min baseline phase was initiated during which the larvae could acclimate and explore the search area. Parallel runs were shifted by 30 s to allow easier handling. Subsequently, the container was placed into the arena center. The larvae were given a time window of up to 15 min to locate and engage with the container*.* A contact was defined by the visual inspection of the experimenter. Accepted were either direct head-on collisions, where larvae bumped into the container and small deformations of the outer cuticle were visible, or cases where larvae ran parallel to the container and made direct head contact during head casts. In each case, a clear physical contact between the container and the larval head was established. Larvae that did not contact the container within the 15 min pre-search interval were excluded from the analysis. They exhibited diverse behaviors: some burrowed into the agarose (which occurred only during baseline and pre-search phases) and remained stationary, others stayed near the arena walls or climbed on the lid, and a third group actively explored the arena without contacting the container. Starting with this first container contact, larvae had 1 min to investigate the container (investigation phase). After that the container was removed, and the larvae were observed for an additional 10 min during the search phase with only the first 5 min being evaluated in the analysis. The following criteria were applied in addition. Firstly, larvae mistakenly perceived as having touched the container were excluded from subsequent data analysis. This happened when larvae passed the initial observation by the experimenter but failed the subsequent video analysis, where higher resolution and frame-by-frame inspection allowed for a more accurate assessment. Secondly, interactions where a larva reached the container from the top via the arena lid were not included in the count. In these cases, the larva could not be tracked with the FIM system because it was hidden by the container's blind spot. We allowed the experiment to continue until the larva reappeared on the agarose plate, at which point tracking resumed. Lastly, larvae that successfully climbed onto the container during the investigation phase were gently removed from it and continued to be observed. The proportion of larvae reaching the target container within the given time frame varied depending on the stimulus. For the different apple juice concentrations, this proportion consistently ranged between 30 and 36%. In the case of olfactory stimuli, the response rates were 26–28% for the various AM concentrations, 27% for 3-OCT and 55% for BA. For the different yeast concentrations, values ranged from 39 to 50%. These results further support our decision to use yeast as a reliable stimulus, as it allows the inclusion of approximately 50% of the larvae tested. Please note that the touch response induced by the chemosensory stimulation is not directly related to the traditional chemosensory response index that is based on the position of larva close to the stimulus but independent of the physical contact (Apostolopoulou et al., 2013; Fishilevich et al., 2005; Louis et al., 2008).

### Additional control experiments

Without container experiments: experiments without container were performed to indicate whether larvae change their behavior from avoiding the center to preferring the center over time. Therefore, larvae were tracked for the maximum duration of the standard protocol (31 min) without any interruption (neither container placing/removing nor opening of the arena lid). In the subsequent data evaluation, the tracking sequence was artificially structured into the four experimental phases.

Empty container experiments: experiments with an empty container were designed to assess the influence of touching the container itself on search behavior. The overall protocol remained consistent, with the only alteration being the placement of an empty container, devoid of any additional stimulus, into the arena.

Odor control experiments: to eliminate the possibility of lingering odor cues causing or influencing centralized search behavior, experiments involving naïve larvae were performed. The experiment started without larvae at the beginning of the baseline phase. Subsequently, a container was introduced into the arena for a duration of 7 min and 16 s (the mean time a larva needed to find the container when using fermented yeast as a chemical stimulus). Afterwards, the container was removed, and a naïve larva was placed in the center of the arena to assess the potential preference for any potentially remaining odor.

Larval cues control experiment: additional experiments with naïve larvae were included to determine the effect of following cues occurring from the larvae itself (e.g. pheromone or mouth hook traces). The experiment was performed as usual with the exception that the larva that interacted with the container was replaced by a naïve animal at the end of the investigation phase. The behavior of this naïve larva was then observed for the search phase only.

### Image processing and tracking of larvae via ImageJ and FIMtrack

Following the recording, the generated image sequences were pre-processed by a self-written ImageJ script (National Institutes of Health, Bethesda, Maryland, https://imagej.net/ij). Regions of interest (ROIs) covering the single arenas were set manually and its center coordinates measured. The script masked the area surrounding ROIs, calculated a median image of the image sequence and determined the difference between the masked and median images to reduce background noise. Finally, the processed data were saved as an image sequence in tiff format. These processed image sequences were then opened with the FIMtrack software, and the larval positions tracked (tracking settings: ‘Gray Threshold’: 100, ‘Min larval size’: 10, ‘Max larval size’: 100). If the software failed to track the larva or split the larval track several times, the ‘Gray Threshold’ was reduced stepwise by a value of 20. Subsequently, the single tracks of an individual larva were stitched together.

### Data evaluation

Tracking data were analyzed via a self-written MATLAB script (The MathWorks Inc. 2021). In a first step FIMtrack data (Risse et al., 2013), center coordinates and information of the experiment (e.g. concentration of the chemical stimulus, time point of container placing) were loaded. Owing to tracking errors, such as reflections or larvae disappearing at the arena edge or container, the detection of larvae failed for some frames (mean=26%, varying between 7.7% and 57.3% dependent on the experiment and experimental phase). If a larva disappeared near the container, we interpreted this as container contact, a conclusion supported by visual inspection. Larvae were clearly visible before and after such events, allowing us to interpolate their positions during undetected frames. This was also used if the larva was not detected in a few frames on the wall of the arena. In each case, it was verified that the larva was assigned to the ‘edge zone’, although the exact *x*-*y* position could not be evaluated. Missing detections which started and ended near the arena edge (>2.13 cm distance to center) were interpolated via a circular path. Gaps occurring at the end of the experiment were interpolated by repeating the last tracked coordinate. All other gaps were interpolated via a straight line. Stepwise other parameters were calculated based on the tracking coordinates.

In the first step, the distance between the larval coordinates and the center was calculated (Pythagoras's theorem) and converted to real size (conversion coefficient: 1 px=0.2673 mm). The distance between consecutive coordinates was computed and cumulated to determine the larval track length. Then, the number of revisits was determined. A revisit was counted if the larva's center of mass crossed the center zone border in the inward direction. The center zone was defined as a center surrounding area with a radius of 0.80 cm. The radius was calculated by summing the radius of the container (0.35 cm), the median larval size (∼0.35 cm) and 0.10 cm for inconsistencies (e.g. larval size variances, imprecise container placement). Furthermore, the time spent in the center zone was calculated and the speed data of the FIMtrack software evaluated. Finally, we quantified the number of stops each larva made during the baseline and search phases. A stop was defined as any instance where the larva moved less than 0.05 mm from its current to the next position. A stop was considered to have ended when the larva moved continuously for at least the next five frames. If two stop phases were separated by five frames or fewer, they were counted as a single stop.

To identify where differences occurred, we refined the analysis. The search arena was divided into seven distance categories (0–4 mm, 4–8 mm, 8–12 mm, 12–16 mm, 16–20 mm, 20–24 mm and >24 mm) and the time spent as well as track length moved in each category was determined. Based on the initial analysis of the position of the larva over time, these initial categories were redefined into four distance categories: center zone (0–8 mm), search zone (8–16 mm), neutral zone (16–24 mm) and edge zone (>24 mm). Furthermore, the mean distances to the arena center within 1 min time intervals were analyzed. Finally, we calculated a search score reflecting the larval behavior with a single parameter. It was calculated by subtracting the time spent in the edge zone from the time spent in a search zone, divided by the total time analyzed (5 min). Positive values displayed a search zone preference, negative values an edge preference. The method of calculation we used is based on the determination of preference values for chemosensory and learning experiments in larvae (Gerber et al., 2009; Michels et al., 2017; Schleyer et al., 2015; Weber et al., 2023; Widmann et al., 2016). This involves determining the difference in the number of larvae located in two differently defined zones on the test plate and relating this to the total number of larvae. This approach is intended to facilitate comparability and standardization across different larval experiments. For the statistical analyzes and visualizations, the data were split into the different experimental phases. Only the first 5 min of the search phase were compared to the baseline. The padcat function of Jos (https://de.mathworks.com/matlabcentral/fileexchange/22909-padcat) was applied for the data evaluation. Significances were visualized using the adapted sigstar function of Campbell (https://github.com/raacampbell/sigstar).

### Statistics

The statistical analysis was conducted with the MATLAB script. We kept the statistics conservative by only performing non-parametric tests. Differences between baseline and search phase were evaluated with the two-sample Wilcoxon signed-rank test. To investigate whether the preference score differed from a random behavior the one-sample Wilcoxon signed-rank test was performed. For the comparison of different test conditions, the Mann–Whitney *U*-test (*n*=2) or Kruskal–Wallis test (*n*>2) were established. A multi-comparison analysis was performed in the case of a significant *P*-value during global testing. Results are visualized in box plots, indicating the median as middle line, 25% and 75% quantiles as box boundaries and minimum/maximum performance indices as whiskers. Significance levels were set to *P≤0.05, **P≤0.01 and ***P<0.001. A detailed statistical evaluation can be found in Tables S1–S3.

### AI tools usage in writing the manuscript

AI tools (such as DeepL and ChatGPT) were employed for grammar, punctuation, language and translation checks. However, the definition of the research question, the methodological approach, the interpretation of the data, the conclusions drawn from the results, and their presentation in the appropriate scientific context were all carried out solely by the authors, without AI assistance. The authors bear full responsibility for the ethical considerations of the research.

## RESULTS

### A paradigm to analyze local search behavior in *Drosophila* larvae

Investigations in insects, such as desert ants (*Cataglyphis fortis*) or adult *Drosophila*, have shown that they can return to a navigational goal even in the absence of external stimuli (Kim and Dickinson, 2017; Muller and Wehner, 1988; Murata et al., 2017). To determine whether *Drosophila* larvae are capable of similar behavior, we developed the larval local search paradigm (Fig. 1A). Following a 1 h period of starvation, individual larvae were positioned in the center of a circular agarose substrate and their movements were recorded in darkness using FIM (Risse et al., 2013). Given the size of the FIM table it was possible to analyze up to seven larvae in parallel, each animal individually in a 6 cm arena on an 8.5 cm agarose patch (Fig. 1B). After observing the larvae's behavior during the initial baseline phase, a Teflon container filled with pure apple juice (100%) was introduced at the arena's center. The larvae had up to 15 min to locate and touch the container during the pre-search phase (Movie 1). They then had 1 min to explore the container, followed by 5 min of observation after the container was removed (search phase). Larvae unable to reach the container within the pre-search phase were excluded from the analysis (numbers are given in the Materials and Methods section). The visualization of the larval tracks revealed that larvae briefly orientate after their placement before departing from the center of the arena (Fig. 1C). Throughout the remaining baseline phase, larvae predominantly stayed in proximity to the edges. In the pre-search phase, larvae explored the arena by circling around the container or moving directly toward it. Of the 55 tested larvae, 20 interacted with the container and were subjected to further observation. While most larvae remained close to the container, a few had already left during the investigation phase. Following the removal of the container, larvae conducted local searches around the previous food spot, with some leaving the center and returning to the edges (Fig. 1C).

**Table 1.** Evaluation of the time larvae spent to find the container

*n*	Yeast concentration			
	25%	50%	75%	100%
1	02:52	04:08	00:31	09:30
2	09:29	00:23	01:39	04:57
3	01:50	14:54	03:44	04:11
4	00:17	14:59	06:56	04:57
5	06:05	12:29	04:06	00:59
6	03:09	01:51	03:17	01:11
7	01:08	02:24	02:47	12:28
8	02:59	03:30	06:02	00:45
9	02:56	08:15	03:05	01:14
10	05:15	01:40	05:42	00:45
11	00:41	09:09	12:01	07:14
12	09:22	03:20	02:26	11:21
13	00:29	13:45	00:32	09:01
14	05:35	10:03	11:02	03:17
15	02:08	–	05:41	00:35
16	02:07	–	02:32	01:19
17	01:35	–	00:26	01:06
18	–	–	01:27	01:13
Mean	3:25	7:12	4:06	4:13

Timings are shown in min:s.

### A parametric analysis of larval local search behavior

Based on the observed changes in larval behavior, we established a processing pipeline to identify and quantify parameters indicative of a local search. Grayscale position plots show the individual temporal larval position during the four phases (color-coded on top) of the experiment (Fig. 2A). The shade of gray corresponds to the larvae's position, ranging from the middle zone through the search and neutral zones to the edge zone, transitioning from dark to light gray (Fig. 2B). Next, we compared the distance of larvae from the center between the baseline and search phases (Fig. 2C). While larvae leave the arena's center within the first minute of the baseline phase and remain, thereafter, close to the edge, they exhibited a more consistent and closer proximity to the center during the search phase (Fig. 2C). Analysis of 1 min time intervals shows that initially larvae start both phases at equivalent distances (Fig. 2D). However, in subsequent 1 min intervals, apart from the fourth, larvae stay closer to the center during the search phase. The larvae do not spend more time in a ‘center zone’ (Figs 2E,F). By dividing the arena radius into seven distinct distance categories we showed that larvae spent more time within a distance of 8–16 mm (from now on called ‘search zone’) from the center and less time within the outermost distance category (from now on called ‘edge zone’) during the search phase (Fig. 2F). This means that larvae circulated around the previous container position.

Despite larvae spending increased time within distinct distance categories (Fig. 2G), this parameter alone does not define a local search behavior. To broaden our analysis, we compared the track length that larvae crawl during baseline and search phases. We found that larvae crawled longer distances during the search phase than during the baseline phase (Fig. 2H). Particularly within the 4–16 mm distance range, larvae traversed considerably longer paths following the removal of the container. Conversely, a shorter track length was observed in the outermost distance category (Fig. 2I,J). The distances covered within the remaining categories remained unaltered. The increased track length observed during the search phase is caused by a higher speed exhibited by the larvae after the presentation of apple juice (Fig. 2K,L). However, the larvae do not spend more time or revisit the center more often during their search than during the baseline phase (Fig. 2M,N). They also stop similarly frequent during baseline and search phase (Fig. 2O). In characterizing the behavior, we also computed a search score, which represents the ratio of the time larvae spent in the search zone compared to the time spent in the outermost region, divided by the total duration of the respective phase (Fig. 2B; for details, see Materials and Methods section). As expected, the larvae exposed to apple juice exhibit a search score that is more positive than that of naïve larvae during the initial baseline phase (Fig. 2P, see Fig. S1 to compare different apple juice concentrations). The combination of all the parameters reveals that, following apple juice presentation, the larvae exhibit extended and more focused movements around the prior container position compared to naïve larvae. Hence, we refer to this locomotion pattern as ‘local search behavior’ of the larvae in the following sections.

### The nature of the chemical stimulus presented exerts varying effects on the local search behavior

Given that apple juice stimulation can induce local search behavior, we subsequently investigated whether olfactory stimuli in general could elicit a similar response. We used the olfactory stimuli amyl acetate (AM, diluted 1:250), benzaldehyde (BA, undiluted) and 3-octanol (3-OCT, undiluted) (Fig. 3). We focused the analysis on the parameters ‘distance to center’, ‘proportion of time spent per distance category’, ‘proportion of track length crawled per distance category’ and ‘search score’ as these allow for a comprehensive description of the behavior.

The introduction of a container filled with AM, a stimulus commonly employed in larval olfactory conditioning (Chen and Gerber, 2014; Scherer et al., 2003; Weber et al., 2023; Widmann et al., 2016), did not evoke local search behavior (Fig. 3A,F,K,P, Movie 2). Neither the distance to center nor the search scores were altered. The AM exposed larvae remained in distances closer to the edge of the arena and avoided the search zone significantly. The same was seen when other AM concentrations (1:1000 and 1:10,000) (Fig. S2) or undiluted 3-OCT (Fig. 3P–T) were used. When undiluted BA was applied, a change in the distance to the center was observed; however, this did not lead to a significant difference in the search score between the baseline and search phases (Fig. 3F,J). In contrast, yeast paste had a more pronounced effect. Larvae reinforced their local search behavior (Fig. 3P,T) reflected by a reduction of their distance to center (Fig. 3P,Q) as well as an extended time spent and track length crawled within distances close to the center (Fig. 3R,S). The experiments demonstrate that odors like apple juice and yeast – typically associated with food sources, unlike those commonly used in learning experiments (AM, 3-OCT and BA) – can elicit local foraging behavior in larvae. Based on the more stable and enhanced effects to induce larval search behavior, we opted to conduct the subsequent experiments using yeast paste.

### Yeast induces local search behavior

Using yeast as a stimulus for larval local search behavior is well supported by ecological and nutritional evidence (Coluccio et al., 2008; Gilbert, 1980; Sang and King, 1961). Adult *Drosophila* prefer yeast-inoculated substrates for oviposition and larvae rely on yeast as a critical nutritional source, particularly for nitrogen, B vitamins, and proteins that are essential for development (Becher et al., 2012; Gibson and Hunter, 2010). Moreover, *Drosophila* can discriminate between yeast strains based on their volatile profiles, indicating a finely tuned sensory system evolved to detect and respond to yeast (Becher et al., 2012; Palanca et al., 2013). Importantly, even in the absence of fruit, fermenting yeast alone is sufficient to drive attraction, oviposition and larval development (Becher et al., 2012). Thus, building on the critical role of yeast, we further explored its impact on larval local search behavior by testing four different yeast concentrations (25%, 50%, 75% and 100%), in addition to fermented yeast and fermented yeast combined with a gustatory stimulus (Fig. 4, Fig. S3). The results displayed a similar outcome for all concentrations. At the onset of the experiment, the larvae departed from the arena's center and positioned themselves closer to the edge. In contrast, during the search phase, they consistently traversed distances near the center, circling around the location where the yeast-filled container had been presented previously (Fig. S3, Movie 3; left). All four groups showed a significant increase in their search scores (Fig. S3). Next, we used fermented yeast as a stimulus (Fig. 4A–E). In an additional experiment, we also covered the lid of a container with fermented yeast to investigate the impact of additional gustatory stimulation (Fig. 4F–J). Larvae exposed to fermented yeast exhibited local search behavior, evident in a noticeable reduction of their distance to the center after interacting with the container compared to the baseline phase (Fig. 4A–C). This effect was even stronger when an additional gustatory stimulus was added to the lid (Fig. 4F–H; *P*=0.003 Table S3). In both cases larvae reduced their time spent and track length crawled within the edge zone (Fig. 4C,H). Larvae that were only stimulated by fermented yeast spent 19% of their time in the center zone and 34% in the search zone. Notably, these larvae showed a significant increase in speed after exposure to fermented yeast (Fig. S4, Movie 3; middle and right). Larvae that had consumed actual food remained within the center zone for more than half of the time (53%) and covered 43% of their total track length in that area (Fig. 4H,I). The larval search score also revealed the behavioral shift of the two groups (Fig. 4E,J). In summary, we see that yeast is a potent stimulus to trigger larval local search behavior, based on the ecological and nutritional importance, fermented yeast seems to be advantageous. The use of additional gustatory stimulation is possible and leads to a centralized search. However, in these experiments it cannot be excluded that the larva contaminates the experimental plate with yeast residues during ingestion.

### Local search behavior is not induced by odor traces or larval residuals and markings

Next, we conducted four additional control experiments to exclude so far not considered confounding effects as the cause of the local search behavior. First, naïve larvae were introduced into a Petri dish for the entire experiment to assess whether their movement patterns change over time in the absence of any additional stimulation (Fig. 5A–E). Throughout the baseline and search phases (only per definition as there is no container) the larvae exhibited identical movement patterns, maintained consistent distances to the center, spent the same time in the same areas and moved the same track lengths therein. As a result, their search scores remained unchanged. Second, we used an empty container without any chemosensory cues inside as a stimulus to test if tactile or mechanosensory stimuli changed larval locomotion in our assay. Indeed, an empty container interaction triggered local search behavior (Fig. 5G–K). After the presentation and removal of the container the larvae remained closer to the center (Fig. 5G–I), spent less time and moved a shorter route length in distance categories closer to the edge. At the same time, the values of these parameters increased at distances closer to the center, especially in the search zone (Fig. 5G,H). The interaction with an empty container also increased the search score (Fig. 5J). In the third experiment, an odor container filled with fermented yeast was positioned on a Petri dish for an average duration of 7:16 min, corresponding to the typical time required for larvae to contact the container. The container was then removed to examine whether residual traces in the agarose or lingering yeast odor cues would influence the search behavior of naïve larvae introduced immediately afterward (Fig. 5K–O). The naïve animals showed baseline-like behavior compared to a control group running in parallel. They remained at greater distances from the center (Fig. 5K) and therefore showed also comparable low search scores (Fig. 5N). The proportion of time spent, and the length of the track covered per distance category was also more similar to that of the baseline phase of the controls (Fig. 5L,M; see Fig. S4). Fourth, we carried out the full protocol but replaced the initial larva with a second naïve animal after the investigation phase. If the first larva had left cues in the arena – such as mechanical or chemical traces – these could potentially guide the naïve larva to search near the previous food location, resulting in behavior that deviates from the original baseline. Our results show that the first larva did not differ in behavior from the second larva. This is in contrast to controls, which showed differences in their local search behavior between the baseline and search phase (Fig. 5P–T). In summary, the findings imply that the larvae do not reduce their distance to the center over time or in response to potential lingering chemical or other larval based stimuli.

### The duration of larval starvation does not affect the local search behavior

In the subsequent experiment, we examined whether the local search behavior is influenced by their feeding state. We compared the local search behavior of well-fed larvae with those that had been starved for 3 and 6 h. Regardless of their starvation status, all tested groups exhibited local search behavior during the search phase (Fig. 6). A comparison between the three experiments and 1 h-starved animals for the search score (Figs 4A–E, 6), time spent in the center zone and number of center revisits during the search phase showed no difference (Table S3; *P*=0.487; *P*=0.841; *P*=0.095, respectively). The proportion of time spent and track length per zone always increased double to triple from baseline to search phase within the search zone (Fig. 6C,D,H,I,M,N). Notably, all test groups displayed significant changes in search scores (see Fig. 6E,J,O). In summary, larval local search behavior appears to occur independently of starvation, probably because of the critical importance of food acquisition during the larval stage, which is the primary feeding phase of the organism.

### Larvae perform local search behavior independent of their genetic background

Based on our previous experiments and to facilitate handling, procedure, and parallelization, we established the following parameters for the standard larval local search experiment: 1 h starvation time, 25% fermented yeast as chemical stimulus, and an agarose concentration of 1.4% to 2.0% for the substrate. We tested agarose concentrations of 0.8%, 1.4% and 2.0% (Fig. S5). In all cases, larvae spent more time and moved farther in the search zone during the search phase compared to the baseline, while spending less time and moving less in the edge zone. This resulted in significantly higher search scores. To minimize larval burrowing into the substrate, we opted for 1.4% or 2.0% agarose, which also facilitated handling and increased the number of larvae making physical contact with the container.

Finally, we evaluated the impact of the genetic background on the local search. In all the experiments described thus far, larvae with a specific mutation in the *white* gene (*w^1118^*) were used. This mutation was chosen due to its widespread use as a genetic background in most available fly lines. Therefore, we have replicated the behavior observed in *w^1118^* mutants using wild-type Canton S (*WT-CS*) larvae. A statistical comparison of the search scores, time spent in the center zone, and number of center revisits during baseline and search phase revealed no significant difference (Table S3; *P*=0.585, *P*=0.266; *P*=0.896, *P*=0.445: *P*=0.675, *P*=0.377; respectively). The larvae of both genotypes resided in the edge zone during the baseline phase in either 66% or 62% of the time (Fig. 7A–C,F–H). Following the interaction with the container, larvae positioned themselves significantly closer to the center and reduced the time spent within this zone (66%→16% for *w^1118^*, 62%→13% for *WT-CS*, Fig. 7C,H), and instead, increased the time spent in the search zone (10%→35% for *w^1118^*, 10%→38% for *WT-CS*, Fig. 7C,H). The same applies for the track length crawled at the edge (Fig. 7D,I). The search scores for both genotypes confirmed the similar behavior of the two groups (Fig. 7E,J). We therefore conclude that the observed behavior in both, *w^1118^* and *WT-CS* larvae, is comparable, enabling future studies on diverse mutant and genetically modified animals.

## DISCUSSION

### *Drosophila* larvae initially show centrophobism/thigmotaxis

We developed the local search paradigm to study larval navigation to a previous food source in an almost featureless habitat. The search behavior is triggered by a physical interaction with an object potentially associated with food, which is subsequently removed. This conclusion is based, first, on the observation that the three tested odors (AM, BA, 3-OCT) do not reliably induce clear larval search behavior in contrast to food-related stimuli such as apple juice or yeast, and second, on the finding that mere physical contact with an empty odor container is sufficient to trigger foraging behavior in larvae (Figs 1, 3–5; Figs S6,S7). Naïve larvae placed in the center of an arena without an additional stimulus leave the middle within 30 s and remain at the edge (Fig. 1). At low agarose concentrations, which the larvae prefer, they can also dig in the middle of the plate to protect themselves from predators and dehydration (Kudow et al., 2019). Adult flies show a similar behavior, as they usually avoid central areas in an arena – either as naïve animals or induced after ether anesthesia (Besson and Martin, 2005; Götz and Biesinger, 1985). This behavior is referred to as centrophobism and/or thigmotaxis and is reminiscent of the open field test in rodents (Archer, 1973) and is also seen in larval and adult zebrafish, as well as in insects such as cockroaches, earwigs, ants and honeybees (Durier and Rivault, 2003; Kalueff et al., 2013; Laurent Salazar et al., 2018; Perttunen, 1952; Schnorr et al., 2012; Stringer et al., 2017; Vázquez and Farina, 2021). In adult *Drosophila*, centrophobism is reduced in learning mutants (e.g. *dunce*) and after MB ablation (Götz and Biesinger, 1985), with intrinsic γ-lobe neurons being particularly important (Besson and Martin, 2005). These neurons are functional from the larval stage and undergo extensive remodeling during metamorphosis (Armstrong et al., 1998; Technau and Heisenberg, 1982; Truman et al., 2023), suggesting that larval local search behavior may depend on the larval MB.

### Yeast is a potent stimulus to trigger local search behavior

Upon the brief presentation of a food stimulus, larval behavior undergoes a notable shift, characterized by the suppression of centrophobism/thigmotaxis and increased movement towards the center where the food source was located (Fig. 1). Complex odors emanating from food sources such as apple juice or yeast initiate local search behavior, in contrast to the single odor AM (Fig. 3), a key aromatic compound found in fruits. AM is attractive to larvae in olfactory choice assays and effective as a conditioned stimulus in classical odor conditioning assays (Chen and Gerber, 2014; Cobb and Dannet, 1994; Pauls et al., 2010; Scherer et al., 2003; Widmann et al., 2016). Variations in yeast concentrations do not significantly impact the intensity of the induced local search behavior (Fig. S3). Yet, larvae display a more centered local search behavior when exposed to fermented yeast odor or when provided with fermented yeast for consumption (Fig. 4). This robust response is somehow expected, given that yeast is the principal food source for both larvae and adults of many *Drosophila* species. Yeast not only supplies essential nutrients but also enhances the availability or mitigates the toxicity of certain compounds, thereby influencing larval growth, survival and body size (Grangeteau et al., 2018). The specific odors associated with *Drosophila* attraction to yeast ferments remain rather unclear. Fermenting *Saccharomyces* species emit volatiles such as ethanol, acids, aldehydes, esters and phenols, which are generally attractive to larvae (Günther and Goddard, 2019; Schumann et al., 2021). The fly's olfactory system detects these via conserved receptors: Or9a/Or92a (acetoin), Or42b (ethyl acetate), Or71a (ethyl phenols) and Or67a/Or85d (phenyl ethanol and phenylethyl acetate) (Becher et al., 2010; Dweck et al., 2015; Stokl et al., 2010). While adults detect glycerol, a sugar alcohol produced by yeast, via the gustatory receptor Gr64a (Wisotsky et al., 2011), this receptor is not expressed in larvae, suggesting they may rely on alternative pathways involving ionotropic receptors Ir76b and Ir25a-expressing neurons (Steck et al., 2018).

### Local search behavior in *Drosophila*

A more detailed parametric analysis revealed that starvation had only a minor influence on search behavior (Fig. 6). It increases a little and becomes more stable after approximately 1 h of starvation but then decreases slightly with prolonged starvation. However, also fed larvae clearly show a local search behavior (Fig. 6). This contrasts with adult *Drosophila* and blowflies that perform a sugar-elicited local search behavior that is dependent on their starvation status (Bell et al., 1985; Dethier, 1957; Kim and Dickinson, 2017; Murata et al., 2017). Adult *Drosophila* are often starved for approximately 1 day in these kinds of experiments. Larvae, owing to their high metabolic rate and rapid growth, may have a generally higher hunger level, which quickly results in negative effects when food is further limited, compounded by the high baseline energy demands of the organism. A similar effect is observed in appetitive classical conditioning experiments, which requires adults, but not larvae, to be food deprived (Gruber et al., 2013; Krashes and Waddell, 2008).

Regarding the behavior itself, differences are evident when comparing adults and larvae. Adults move in short, straight segments interrupted by saccadic turns, often returning to the exact location of a distant food source (Behbahani et al., 2021; Corfas et al., 2019; Kim and Dickinson, 2017; Murata et al., 2017). In contrast, larvae tend to follow a rough circular path around the initial food stimulus and often do not return to the exact starting point (Figs 1,2). This difference may stem from distinct strategies each must employ. Recent experiments show that flies possess head direction cells in the CX that help maintain an absolute sense of orientation (Seelig and Jayaraman, 2015). Such cells have not been identified in larvae, and the reconstruction of the larval connectome does not yet reveal any obvious corresponding wiring pattern (Winding et al., 2023). Consequently, it is likely that the simpler cellular structure of the larval brain does not support this function, resulting in less precise and less positionally accurate local search behavior.

### Larval search strategies

What search strategy could the larva use? We would like to propose four different options in ascending complexity: (1) simple changes of the motor pattern, (2) sensory taxis, (3) place memory and (4) idiothetic path integration. A straightforward explanation for local foraging behavior could be that the brief food stimulus shifts the larvae's movement pattern from an exploratory mode to a more local one. A similar fundamental shift in foraging behavior is reported for the foraging gene; larvae with the rover allele travel longer distances than those with the sitter allele (Osborne et al., 1997; Sokolowski, 1980). These different foraging patterns are achieved by altering the speed and frequency of pauses and turns (Gomez-Marin et al., 2011; Sokolowski, 1980). In the simplest scenario, the yeast stimulus slows down the larvae's movement and increases the number of pauses for several minutes, keeping them closer to the center of the arena (Wosniack et al., 2022). However, the larvae in our assay behave in the opposite way. During the search, they increase their basic speed in response to fermented yeast (Fig. 2K). Therefore, other strategies must be present.

Larvae might also receive sensory cues during its local search that cause it to turn back toward the center as it moves away from it, as the gradient of sensory stimuli decreases. Consequently, the larva engages directed movement, or taxis. But what could this sensory stimulus be? Larvae possess a simple visual system that is incapable of perceiving infrared light, which we utilized in our experiments, allowing us to rule out visual stimuli (Humberg et al., 2018; Keene and Sprecher, 2012; Sprecher et al., 2011). Likewise, no specific acoustic or tactile stimuli were present in the experimental setup that the larva could use. We also minimized the potential influence of taste by enclosing the yeast and apple juice in a container with a perforated lid so that no food residuals could get onto the agarose. Therefore, we propose that the only remaining means for the larva to orient itself is through olfactory cues. This could be due to lingering residues of yeast and apple juice odors, or pheromones that the larvae use to mark the location of the food source (Mast et al., 2014). However, when naïve larvae are placed on test plates where previously a larva and/or fermented yeast was presented, they do not exhibit local search behavior (Fig. 5). In addition, the odor stimulus from the container is very weak. For apple juice, only a good third of the larvae even found the container in the arena. Likewise, the direct contact time is limited to only 1 min, so it is unlikely that this time is sufficient to set a pheromone signal. Therefore, this explanation does not seem completely convincing either.

It is also possible that the larvae develop a spatial memory for the position of the stimulus. Social insects like bees and ants use memorized visual cues to maintain direction (alignment image-matching) and navigate to familiar locations (positional image-matching) (Collett et al., 2013). Similarly, adult *Drosophila* can recognize and remember a visual environment in a heat maze test to find a cool spot in a heated arena (Ofstad et al., 2011). They can also recall the direction of a visual stimulus even when it is no longer present and remember the spatial location of a cold spot in a warm environment using mechanosensory cues (Neuser et al., 2008; Ostrowski et al., 2015; Wustmann et al., 1996). Thus, larvae might employ a comparable form of memory. Mechanosensory information might be at play. Therefore, one could now investigate which mechanosensory neurons or brain areas are crucial for the local search behavior.

Finally, larvae may rely on idiothetic cues to relocate previously visited feeding sites, as seen in adult flies and other insects (Corfas et al., 2019; Kim and Dickinson, 2017; Muller and Wehner, 1988; Wittlinger et al., 2006). Although larvae lack a CX, which is crucial for tracking distances and orientations to determine the current position in adults, they possess sensory neurons to detect proprioceptive feedback (Agrawal and Tuthill, 2022; Greaney et al., 2023; Richter et al., 2024; Vaadia et al., 2019; Winding et al., 2023). Yet, larval locomotion is highly stereotyped, characterized by rhythmic peristaltic waves for crawling and unilateral contractions of one side of the body for head casting and turning (Berni et al., 2012; Dixit et al., 2008; Fox et al., 2006; Gjorgjieva et al., 2013; Heckscher et al., 2012; Lahiri et al., 2011). This rhythmic movement, consisting of runs and turns, can be generated independently of sensory feedback and descending input from the brain, suggesting the presence of a central pattern generator network in the thoracic and abdominal segments (Berni et al., 2012; Hughes and Thomas, 2007; Suster and Bate, 2002). This raises the possibility of a basic crawl counter mechanism, similar in concept to a step counter observed in ants.

With this study providing a comprehensive parametric description of the larva's local search behavior, it should now be possible to decipher the underlying neuronal network in the next steps of our research. Since this foraging behavior is not unique to *Drosophila* larvae, it will also be intriguing to determine whether a rudimentary larval CX exists, or whether the larva has developed a different coding strategy.

## Supplementary Material

10.1242/jexbio.249913_sup1 Supplementary information
