# Supplementary material for: Finding a path: local search behavior of Drosophila larvae
Source: J Exp Biol. 2025 Nov 13;228(22):jeb249913. doi: 10.1242/jeb.249913 (PMC12669832; doi:10.1242/jeb.249913)
Supplement: Supplementary information [file jexbio-228-249913-s1.pdf]

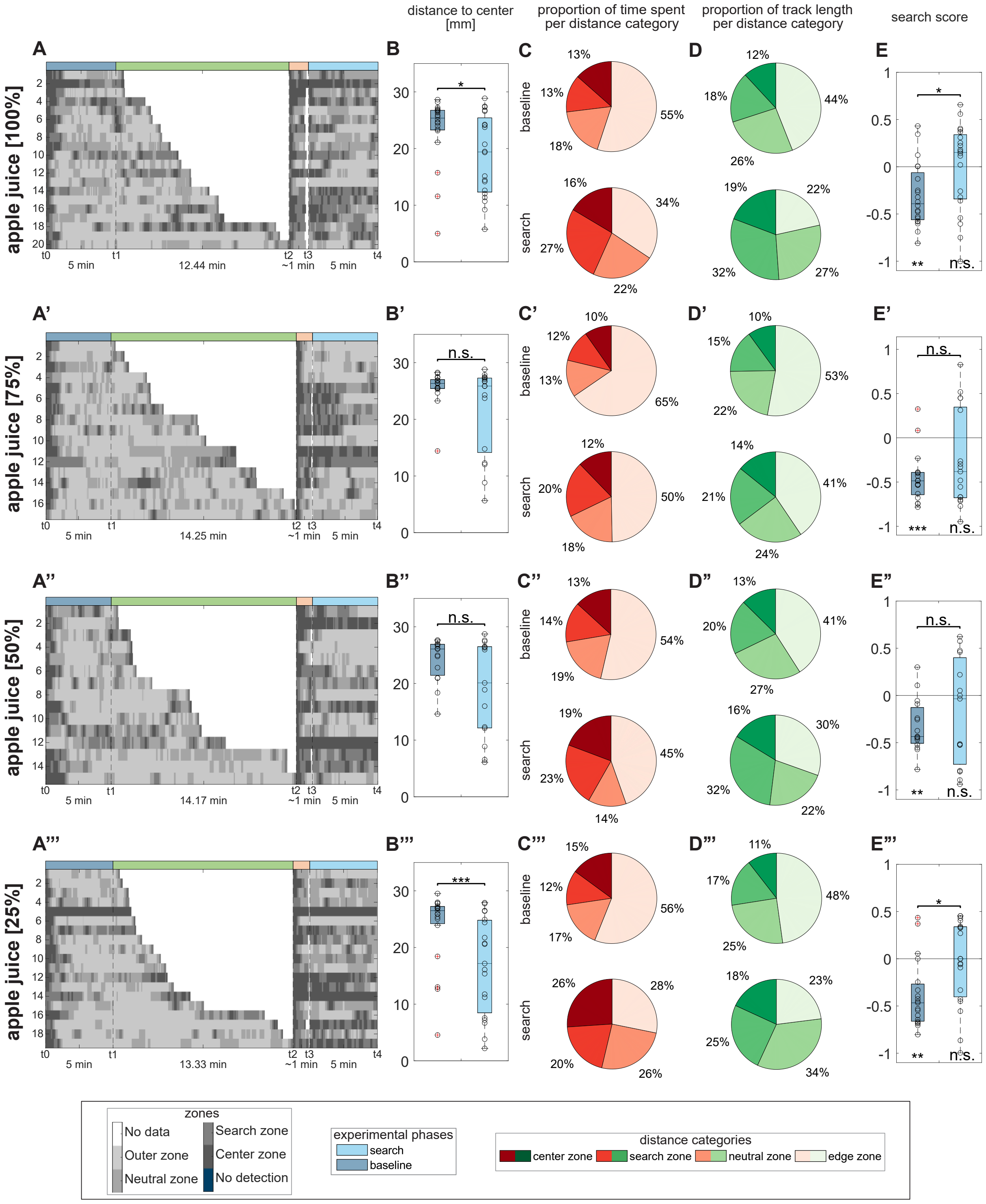

Fig. S1. Effect of different apple juice concentrations on the local search behavior.

Larvae were tested via the larval local search paradigm to evaluate the influence of different apple juice concentrations, 25%, 50%, 75% and 100%, on the larval behavior.

(A) Grey-scale position plots for each experiment.

(B) Distance to center – boxplot. Larvae exposed to low (25%), and high (100%) concentrations of apple juice remained closer to the center after the container interaction (B: p=0.019; B''': p<0.001). In contrast the distance to center after a presentation of 50% and 75% apple juice is unaffected (B': p=0.113; B'': p=0.064).

(C) Proportion of time spent per distance category. Pie charts represent the respective proportion counterclockwise from center (dark red) to edge (beige).

(D) Proportion of track length crawled per distance category. Pie charts represent the respective proportion counterclockwise from center (darkest green) to edge (lightest green).

(E) Search score. All test groups preferred the edge during the *baseline* and showed a neutral behavior after the container interaction (E: p_{base}=0.003, p_{search}=0.852; E': p_{base}<0.001, p_{search}=0.093; E'': p_{base}=0.002, p_{search}=0.241; E''': p_{base}=0.001, p_{search}=0.795). Only larvae exposed to 25% and 100% apple juice increased their search score significantly (E: p=0.014; E': p=0.163; E'': p=0.489; E''': p=0.016).

The larvae were tested after one-hour starvation time. To compare other apple juice concentrations with apple juice (100%), we present parts of the data of Fig. 2 here again. For the statistical evaluation the one-sample and two-sample Wilcoxon signed-rank test were performed. *p≤0.05, **p≤0.01, ***p<0.001.

Journal of Experimental Biology • Supplementary information

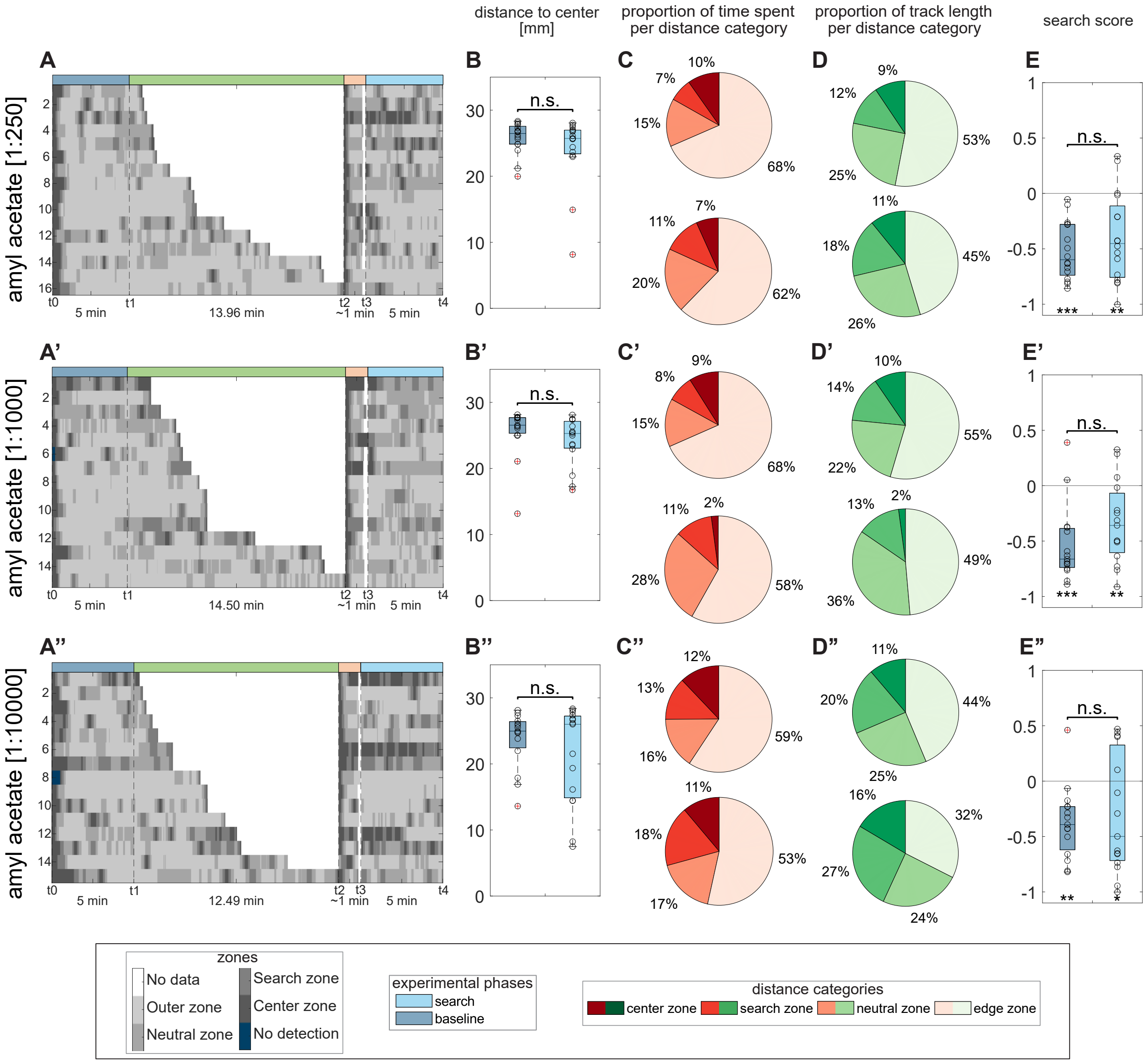

Fig. S2. Impact of the AM concentration on the larval behavior. Larvae were tested via the local search paradigm to examine the effect of different AM concentrations (1:250, 1:1000, 1:10000).

(A) Grey-scale position plots for each experiment.

(B) Distance to center – boxplot. None of the test groups lower their distance to center after the interaction with the container (B: p=0.079, B': p=0.454, B'': p=0.720).

(C) Proportion of time spent per distance category. Pie charts represent the respective proportion counterclockwise from center (dark red) to edge (beige).

(D) Proportion of track length crawled per distance category. Pie charts represent the respective proportion counterclockwise from center (darkest green) to edge (lightest green).

(E) Search score. All larvae prefer the edge during *baseline* and *search phase* (E: $p_{base|search}$<0.001|0.002, E': $p_{base|search}$<0.001|0.007, E'': $p_{base|search}$=0.003|0.035). Changes in the search score are not significant (E: p=0.234, E': p=0.135, E'': p=0.934).

Larvae were tested after one-hour starvation. For the statistical evaluation the one-sample and two-sample Wilcoxon signed-rank test were performed. *p≤0.05, **p≤0.01, ***p<0.001.

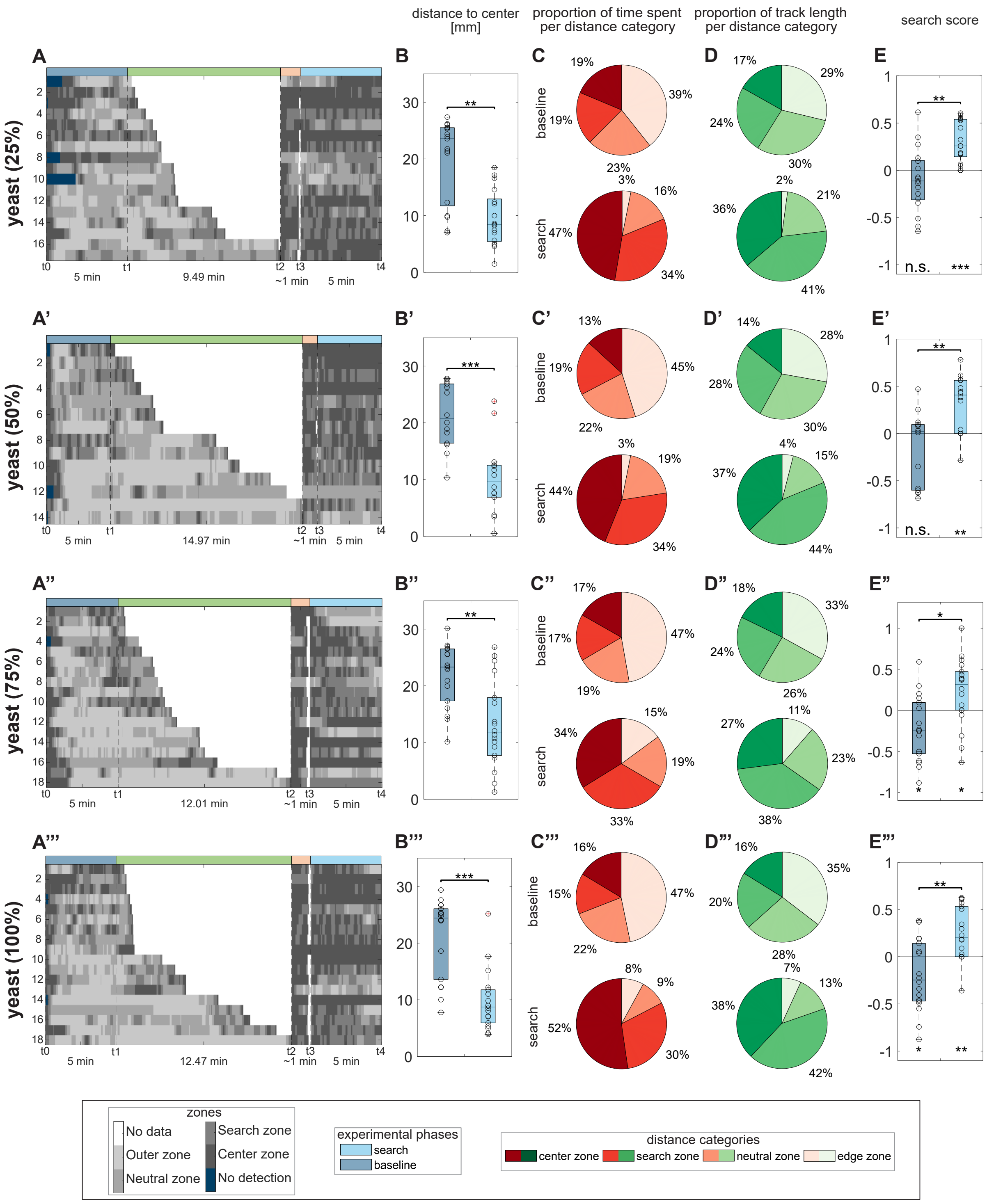

Fig. S3. Influence of the yeast on the search behavior. Larvae were tested via the local search paradigm to examine the effect of different yeast concentrations 25%, 50%, 75% and 100%.

(A) Grey-scale position plots for each experiment.

(B) Distance to center – boxplot. All test groups reduced their distance to center significantly after the yeast presentation (B: p=0.002, B': p<0.001, B'': p=0.006, B''': p<0.001).

(C) Proportion of time spent per distance category. Pie charts represent the respective proportion counterclockwise from the center (dark red) to the edge (beige).

(D) Proportion of track length crawled per distance category. Pie charts represent the respective proportion counterclockwise from the center (darkest green) to the edge (lightest green).

Journal of Experimental Biology • Supplementary information

(E) Search score. All larvae avoided or behaved neutrally during the *baseline phase* and preferred the search zone after the yeast presentation (E: p_{base}=0.210, p_{search}<0.001; E': p_{base}=0.356, p_{search}=0.003; E'': p_{base}=0.025, p_{search}=0.049; E''': p_{base}=0.039, p_{search}=0.003).

Each group increased their search score significantly (E: p=0.003, E': p=0.005, E'': p=0.018, E''': p=0.001).

All larvae were tested after one-hour starvation time. For the statistical evaluation the one-sample and two-sample Wilcoxon signed-rank test were performed. *p≤0.05, **p≤0.01, ***p<0.001.

Journal of Experimental Biology • Supplementary information

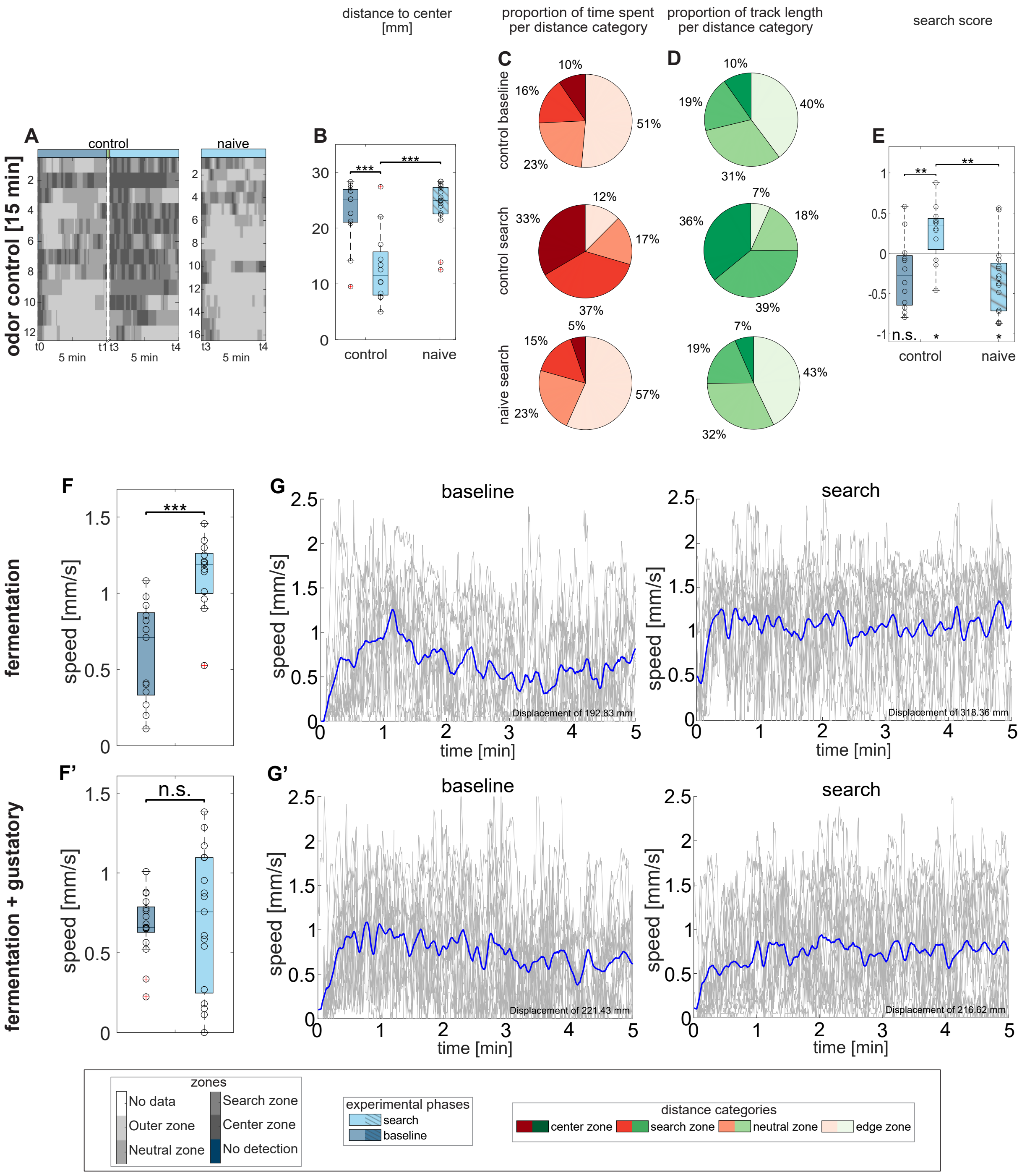

Fig. S4. Impact of potentially lingering chemical cues on the local search behavior and the impact of fermentation products on the larval speed. Shown are the results of the larval local search paradigm for naïve larvae placed into the search arena after 15 min of odor evaporation compared to a parallel running behavioral control group (A-F) and the comparison of speed for larvae tested before and after the presentation of fermented yeast with or without a gustatory stimulus.

(A) Grey-scale plots for each experiment.

(B) Distance to center – boxplot. Naïve larvae performed a *baseline*-like behavior

Journal of Experimental Biology • Supplementary information

characterized by a high distance to center ($p=0.763$). Both, naïve larvae, and the behavioral controls *baseline phase*, differ significantly from the *search phase* ($p_{naïve-search}<0.001$, $p_{base-search}=<0.001$).

(C) Proportion of time spent per distance category. Pie charts represent the respective proportion counterclockwise from the center (dark red) to the edge (beige).

(D) Proportion of track length crawled per distance category. Pie charts represent the respective proportion counterclockwise from the center (darkest green) to the edge (lightest greens).

(E) Search score. Naïve larvae preferred the edge zone during their test phase while the behavioral control larvae were neutral during the *baseline phase* and preferred the search zone during their *search phase* ($p_{naïve}=0.015$, $p_{base}=0.064$, $p_{search}=0.043$). The behavior of the naïve animals is indistinguishable from the controls *baseline* but differs from their *search phase* ($p_{naïve-base}=0.562$, $p_{naïve-search}=0.003$, $p_{base-search}=0.005$).

(F) Speed – boxplot. Larvae exposed to the fermented yeast shows a higher speed after the presentation of the stimulus than before (F: $p=0.001$). In contrast, the speed does not rise significantly if the larvae had an additional gustatory stimulus (F': $p=0.622$).

(G) Speed - progression over time. The graphs display the mean speed averaged over ten frames of the test group (blue line) as well as the individual speed (grey lines) over time.

The larvae were tested after one-hour starvation time. For the statistical evaluation the one-sample and two-sample Wilcoxon signed-rank test were performed. *$p\leq0.05$, **$p\leq0.01$, ***$p<0.001$.

Journal of Experimental Biology • Supplementary information

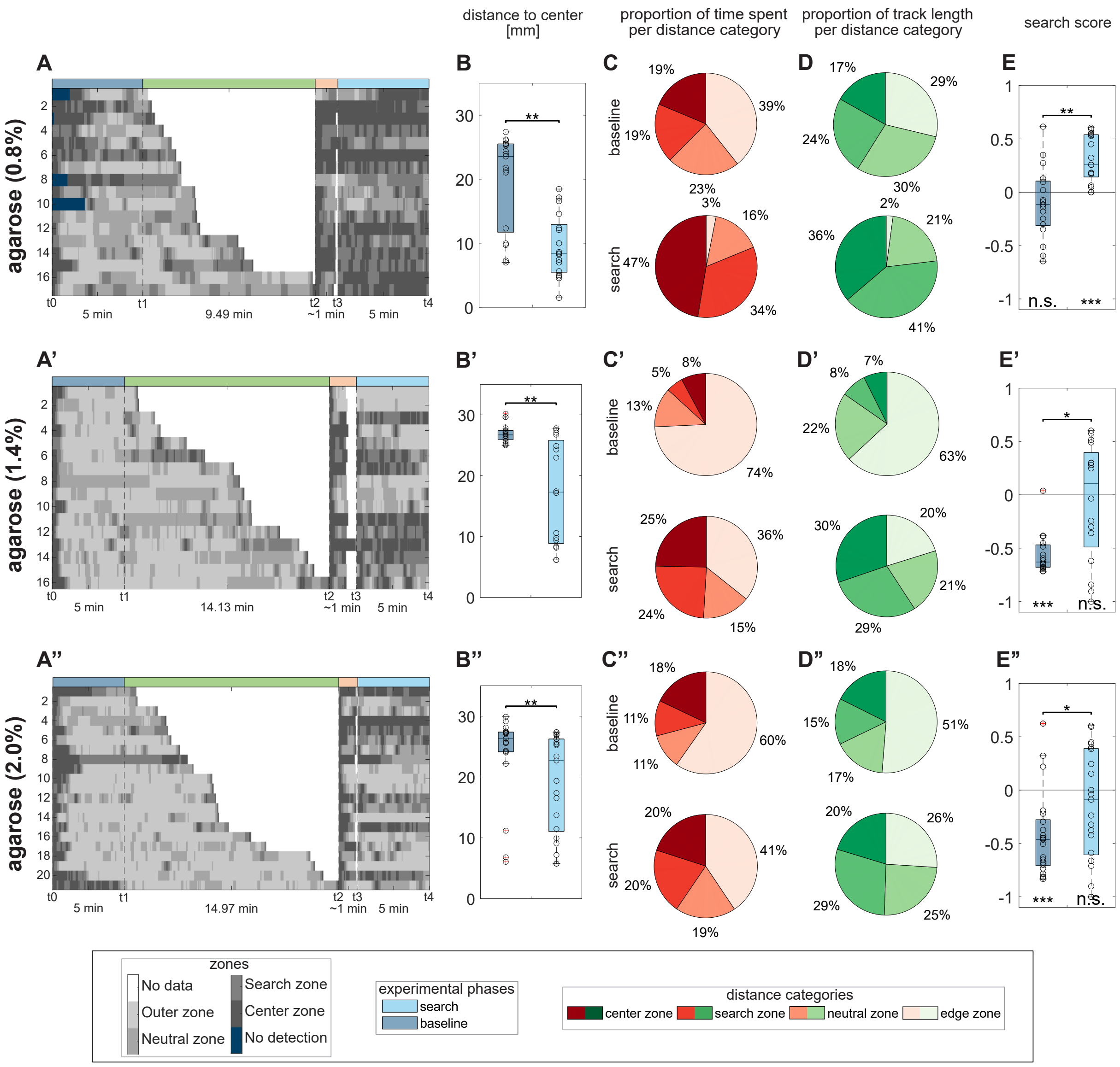

Fig. S5. Rising agarose concentrations decrease the local search. Larvae were tested via the local search paradigm to examine the effect of different agarose concentrations (0.8%, 1.4% and 2.0%).

(A) Grey-scale position plots for each experiment.

(B) Distance to center – boxplot. All test groups lowered their distance to center significantly after the container interaction (B: p=0.002, B': p=0.003, B'': p=0.006).

(C) Proportion of time spent per distance category. Pie charts represent the respective proportion counterclockwise from center (dark red) to edge (beige).

(D) Proportion of track length crawled per distance category. Pie charts represent the respective proportion counterclockwise from center (darkest green) to edge (lightest green).

Journal of Experimental Biology • Supplementary information

(E) Search score. Larvae tested on 0.8% agarose plates neither preferred nor avoided the search zone before the stimulus presentation (E: p=0.210) but showed a preference towards it after the interaction (E: p=0.003). The remaining test groups avoided the search zone during the *baseline phase* (E': p<0.001; E'': p<0.001) and shifted into a neutral one after the stimulus presentation (E': p=0.717; E'': p=0.296). All larvae increased their search score significantly (E: p=0.003, E': p=0.011, E'': p=0.042).

Larvae were tested after one-hour starvation using yeast (25%). Notice that the data of the 0.8% agarose test group are taken from the yeast (25%) experiment and were therefore not run in parallel to the other agarose concentrations. For the statistical evaluation the one-sample and two-sample Wilcoxon signed-rank test were performed. *p≤0.05, **p≤0.01, ***p<0.001.

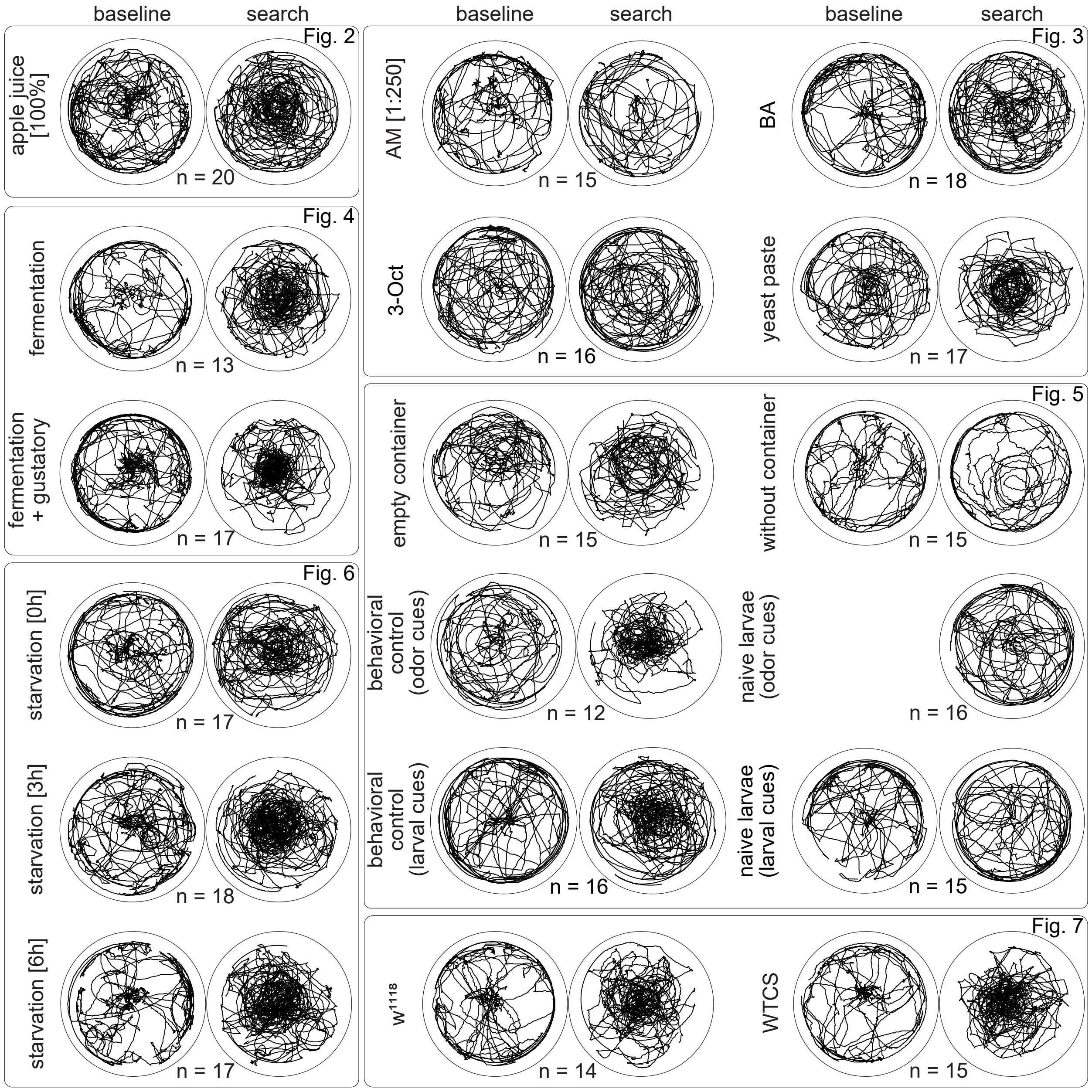

Fig. S6. Larval walking trajectories during baseline and search phase. The visualization displays the larval behavior under different conditions.

Journal of Experimental Biology • Supplementary information

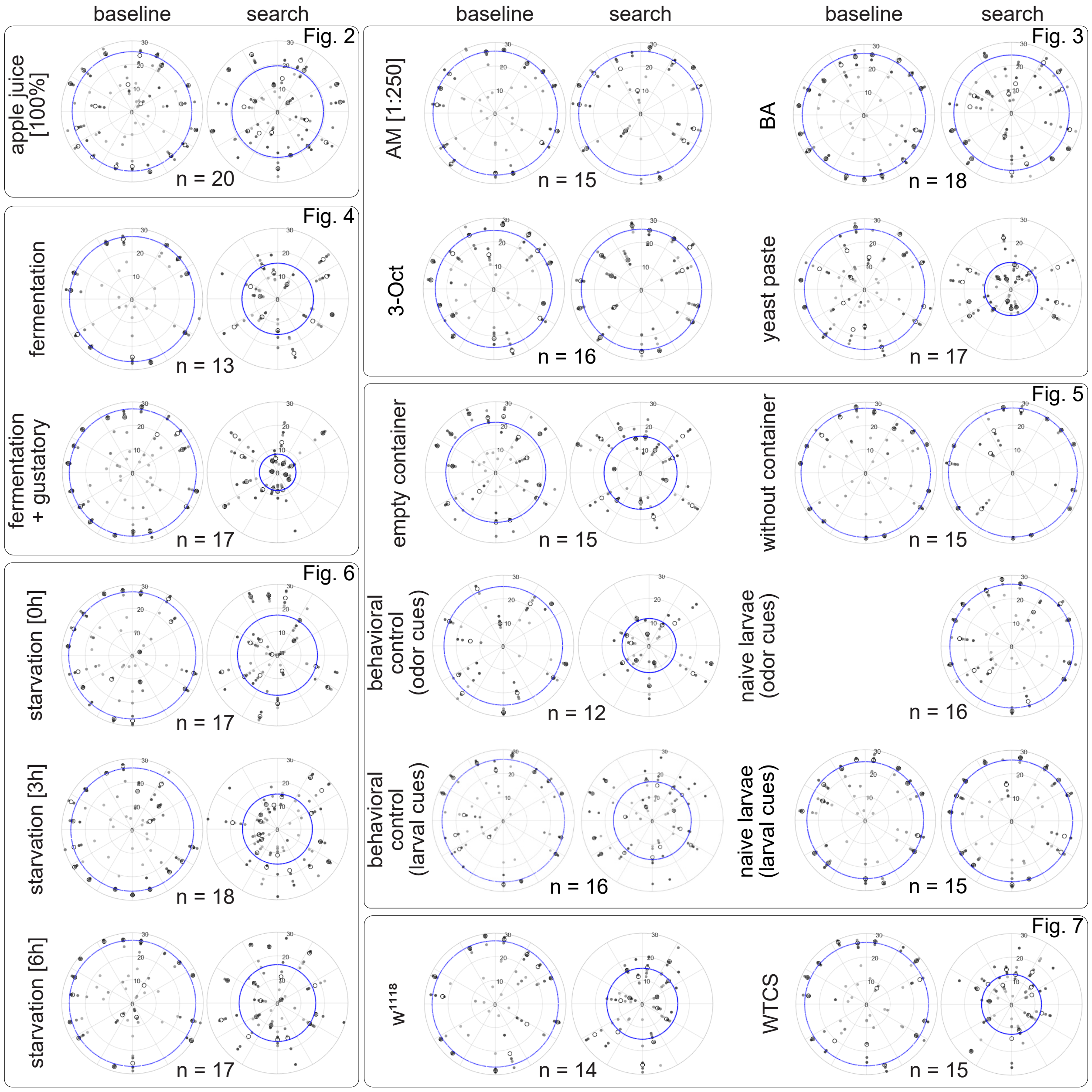

Fig. S7. Larval 2D scatter plots for baseline and search phases. Displayed are the individual larval distances to center under different conditions. Each line emerging from the center represents a single larva and each filled dot the mean larval distance within a one-minute time interval (decreasing brightness). Circles show the larval median distance during the entire phase. The blue circle represents the median distance of the entire test group.

Journal of Experimental Biology • Supplementary information

Table S1. Statistical evaluation of the larval local search paradigm. The table visualizes the p-values and medians from the two parameters distance to center and search score of the performed experiments. Significant p-values are highlighted in bold.

**test group / comparisons**	**distance to center**		**search score**			**displayed in figure**
	p-values two-sample Wilcoxon signed rank or Mann Whitney U test (median: baseline | search [mm])		p-values two-sample Wilcoxon signed rank or Mann Whitney U test (median: baseline | search)		p-values one-sample Wilcoxon signed rank test (baseline | search)	
apple juice (100%)	**0.019**	(25.36 | 19.38)	**0.014**	(-0.392 | 0.152)	**0.003** | 0.852	2/S1
empty	**0.022**	(21.25 | 15.51)	**0.005**	(-0.235 | 0.347)	0.064 | 0.073	5
amyl acetate (1:250)	0.934	(26.34 | 26.32)	0.793	(-0.459 | -0.533)	**< 0.001** | **0.005**	3
yeast paste	**0.002**	(25.58 | 11.27)	**0.001**	(-0.337 | 0.125)	**0.031** | **0.007**	3
yeast (25%)	**0.002**	(23.56 | 8.42)	**0.003**	(-0.113 | 0.257)	0.210 | **< 0.001**	S2/S5
yeast (50%)	**< 0.001**	(20.70 | 9.70)	**0.005**	(0.021 | 0.408)	0.356 | **0.003**	S2
yeast (75%)	**0.007**	(23.31 | 11.70)	**0.018**	(-0.250 | 0.316)	**0.025** | **0.049**	S2
yeast (100%)	**< 0.001**	(24.42 | 8.73)	**0.001**	(-0.246 | 0.206)	**0.039** | **0.003**	S2
agarose (1.4%)	**0.003**	(26.72 | 17.27)	**0.011**	(-0.630 | 0.108)	**< 0.001** | 0.717	S3
agarose (2.0%)	**0.006**	(26.30 | 22.72)	**0.042**	(-0.467 | -0.090)	**< 0.001** | 0.296	S3
fermentation	**< 0.001**	(26.56 | 15.17)	**< 0.001**	(-0.676 | 0.329)	**< 0.001** | 0.080	4
fermentation + gustatory	**< 0.001**	(27.02 | 7.76)	**< 0.001**	(-0.471 | 0.062)	**< 0.001** | 0.119	4
without container	0.340	(27.42 | 27.34)	0.330	(-0.651 | -0.938)	**< 0.001** | **< 0.001**	5
starvation (0h)	**< 0.001**	(27.01 | 16.99)	**0.001**	(-0.571 | 0.062)	**0.003** | 0.847	6
starvation (3h)	**0.006**	(26.11 | 14.85)	**0.003**	(-0.538 | 0.164)	**0.003** | **0.039**	6
starvation (6h)	**0.019**	(26.79 | 16.29)	**0.002**	(-0.638 | 0.038)	**< 0.001** | 0.196	6
w^{1118}	**< 0.001**	(26.74 | 15.10)	**< 0.001**	(-0.629 | 0.281)	**< 0.001** | **0.025**	7
WTCS	**< 0.001**	(26.22 | 12.71)	**< 0.001**	(-0.437| 0.376)	**< 0.001** | **0.010**	7
control (baseline) | naive	0.593	(25.19 | 26.21)	0.341	(-0.279| -0.538)	(0.064 | **0.003**)	5
control (search) | naive	**< 0.001**	(11.46 | 26.21)	**< 0.001**	(0.341 | -0.538)	(**0.043** | **0.003**)	5
control (baseline) | control (search)	**< 0.001**	(25.19 | 11.46)	**0.005**	(-0.279 | 0.341)	(0.064 | **0.043**)	5
apple juice (75%)	0.113	(26.34 | 25.87)	0.163	(-0.486 | -0.381)	**< 0.001** | 0.093	S1
apple juice (50%)	0.064	(26.08 | 20.09)	0.489	(-0.436 | -0.033)	**0.002** | 0.241	S1

Journal of Experimental Biology • Supplementary information

apple juice (25%)	**< 0.001**	(26.50 | 17.18)	**0.016**	(-0.466 | 0.000)	**0.001** | 0.795	S1
control (baseline) | naive	0.763	(25.19 | 24.96)	0.562	(-0.279 | -0.343)	(0.064 | **0.015**)	S4
control (search) | naive	**< 0.001**	(11.46 | 24.96)	**0.003**	(0.341 | -0.343)	(**0.043** | **0.015**)	S4
control (baseline) | control (search)	**< 0.001**	(25.19 | 11.46)	**0.005**	(-0.279 | 0.341)	(0.064 | **0.043**)	S4
amyl acetate (1:250)	0.079	(26.47 | 25.72)	0.234	(-0.599 | -0.452)	(**< 0.001** | **0.002**)	S5
amyl acetate (1:1000)	0.454	(26.55 | 25.29)	0.135	(-0.663 | -0.359)	(**< 0.001** | **0.007**)	S5
amyl acetate (1:10000)	0.720	(24.97 | 26.00)	0.934	(-0.392 | -0.499)	(**0.003** | **0.035**)	S5
3-octanol	0.756	(24.86 | 25.60)	0.796	(-0.346 | -0.490)	(**0.010** | **0.049**)	3
benzaldehyde	**0.018**	(26.17 | 24.54)	0.145	(-0.628 | -0.250)	(**< 0.001** | 0.078)	3
control larval cues (naive)	0.359	(24.91 | 25.83)	0.359	(-0.309 | -0.499)	(**< 0.001** |**< 0.001**)	5
control larval cues (control)	**0.008**	(25.91 | 16.46)	**0.008**	(-0.578 | 0.185)	(**0.001** | 0.642)	5

Journal of Experimental Biology • Supplementary information

Table S2. Statistical evaluation of the apple juice (100%) experiment. Visualized are the p-values and medians of the different parameters evaluated in Fig.2. Significant p-values are highlighted in bold.

	p-values two-sample Wilcoxon signed rank test (median baseline | search)	
**Fig.2D (distance [mm] per time interval)**		
1	0.135	(12.05 | 12.05)
2	**0.004**	(24.54 | 15.00)
3	**0.003**	(25.05 | 20.94)
4	0.126	(24.83 | 21.37)
5	**0.037**	(25.05 | 19.78)
**Fig.2F (time spent per distance category [s])**		
0-4 mm	0.446	(6.25 | 4.25)
4-8 mm	0.167	(25.25 | 24.50)
8-12 mm	**0.038**	(16.00 | 48.75)
12-16 mm	**0.018**	(12.50 | 31.00)
16-20 mm	0.117	(14.00 | 21.25)
20-24 mm	0.809	(31.25 | 31.75)
> 24 mm	**0.014**	(190.00 | 81.50)
**Fig.2H (track length [cm])**		
	**0.017**	(24.09 | 27.85)
**Fig.2I (track length per distance category [cm])**		
0-4 mm	0.396	(0.43 | 0.51)
4-8 mm	**0.040**	(1.83 | 2.85)
8-12 mm	**0.023**	(1.67 | 4.77)
12-16 mm	**0.015**	(1.46 | 3.43)
16-20 mm	0.145	(1.51 | 2.34)
20-24 mm	0.841	(3.31 | 2.94)
> 24 mm	**0.010**	(12.03 | 4.27)
**Fig.2K (speed [mm s^{-1}])**		
	0.**001**	(0.64 | 0.92)
**Fig.2M (time spent in center zone [min])**		
	0.380	(0.58 | 0.41)
**Fig.2N (centre revisits, [crv])**		
	0.092	(2.0 | 2.5)
**Fig.2O (number of stops)**		
	0.058	(11 | 8)

Journal of Experimental Biology • Supplementary information

Table S3. Statistical evaluation of the parameters search score, time spent in center zone and number of center revisits. Visualized are the p-values for comparing different yeast, starvation, and genotype experiments. Significant p-values are highlighted in bold.

Compared groups	Search score	Time spent in center zone (min)	Number of center revisits
**yeast paste, fermentation, fermentation + gustatory**			
2-sample Wilcoxon:			
• yeast paste	**0,001** (-0.337 | 0.125)	0.076 (0.67 | 0.96)	0.175 (2 | 3)
• fermentation	**< 0.001** (-0.676 | 0.329)	0.273 (0.51 | 0.54)	**0.017** (1 | 4)
• fermentation + gustatory	**< 0.001** (-0.471 | 0.062)	**0.003** (0.58 | 2.83)	0.109 (2 | 5)
KW:	**0.042** | 0.615	0.599 | 0.058	0.150 | 0.292
mc:			
• yeast paste / fermentation	**0.039** | -		
• yeast paste / gustatory	0.400 | -		
• fermentation / gustatory	0.826 | -		
**starvation**			
2-sample Wilcoxon:			
• 0h	**0.001** (-0.571 | 0.062)	0.255 (0.51 | 0.68)	0.342 (2 | 3)
• 1h	**< 0.001** (-0.676 | 0.329)	0.273 (0.51 | 0.54)	**0.017** (1 | 4)
• 3h	**0.003** (-0.538 | 0.164)	0.102 (0.63 | 0.88)	**0.002** (2 | 5)
• 6h	**0.002** (-0.638 | 0.038)	0.492 (0.43 | 0.84)	**0.012** (1 | 5)
KW:	0.574 | 0.487	0.918 | 0.841	0.452 | 0.095
**w^{1118}/WTCS**			
2-sample Wilcoxon:			
• W^{1118}	**< 0.001** (-0.629 | 0.281)	**0.035** (0.58 | 1.53)	**0.025** (1 | 4)
• WTCS	**< 0.001** (-0.437| 0.376)	0.131 (0.72 | 0.87)	**0.016** (2 | 3.5)
MWU:	0.585 | 0.266	0.896 | 0.445	0.675 | 0.377

Journal of Experimental Biology • Supplementary information

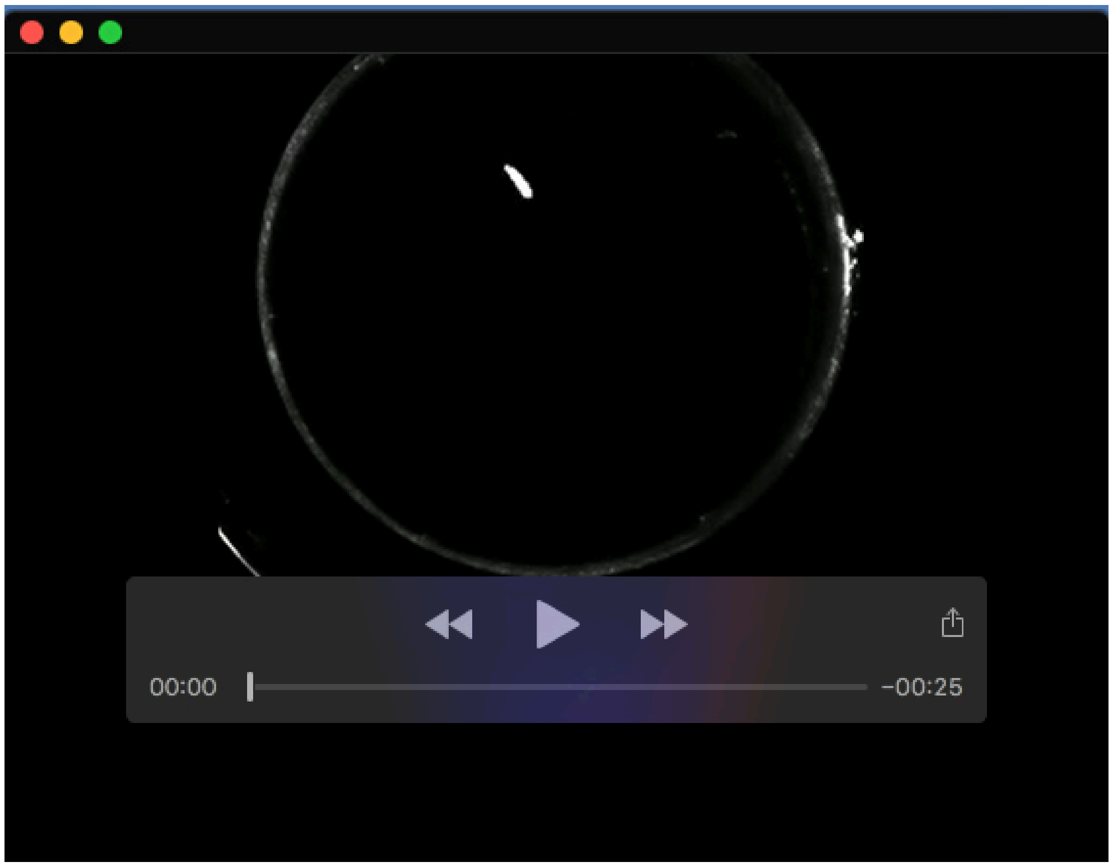

Movie 1. Larval behavior before and after the presentation of 100% apple juice. The video shows the behavior of a single larva during the entire local search paradigm.
The video was recorded with two frames per second and playback is sped up by a factor of ten.

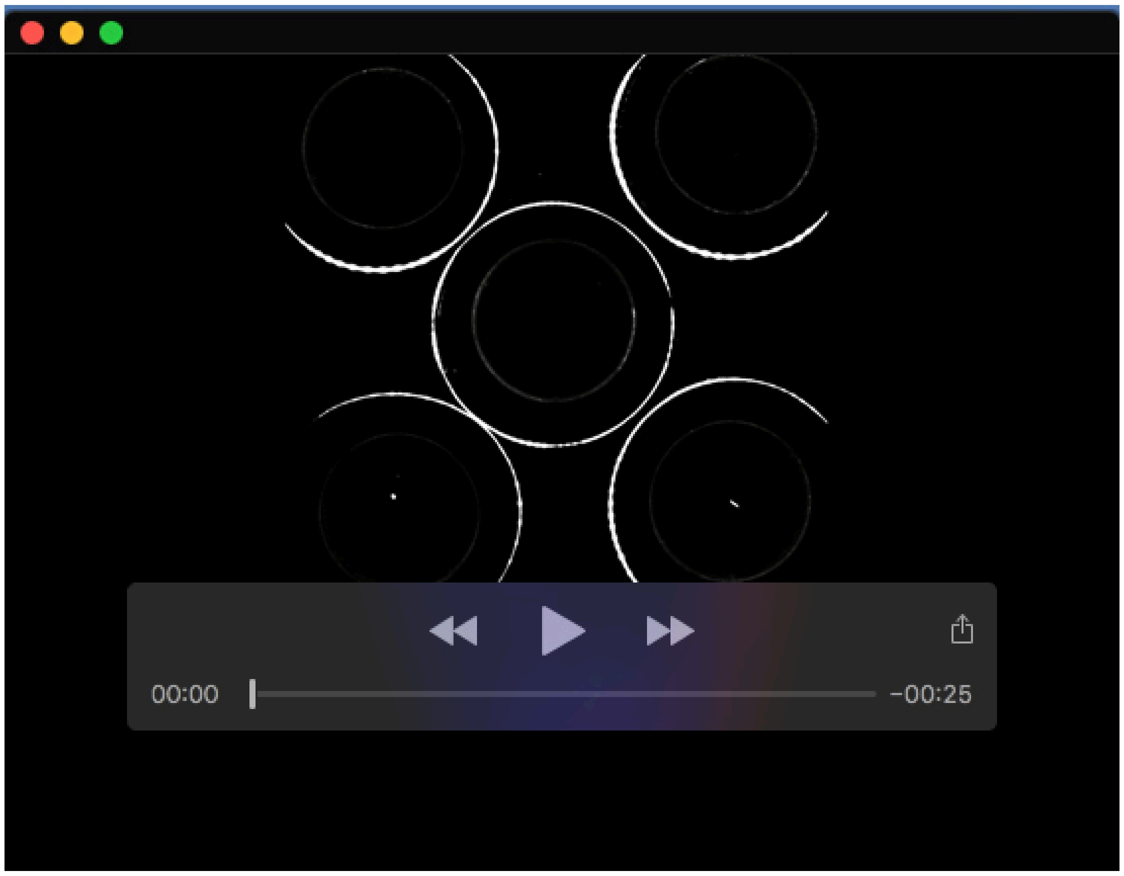

Movie 2. Larval behavior before and after the presentation of amyl acetate. The video shows the behavior of four larvae during the entire local search paradigm. The video was recorded with two frames per second and playback is sped up by a factor of ten.

Journal of Experimental Biology • Supplementary information

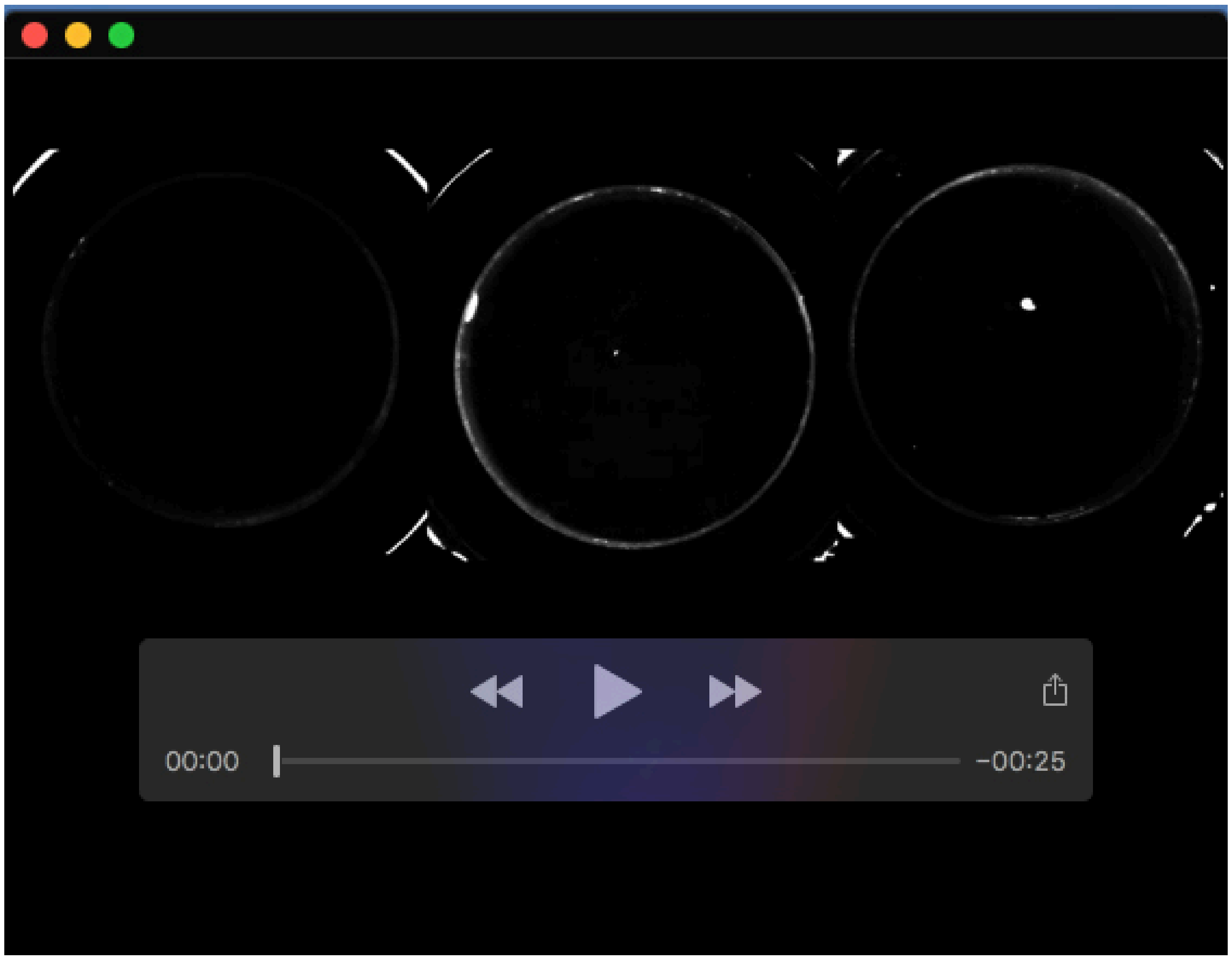

Movie 3. Larval behavior before and after the presentation of a yeast paste (25%) and fermented yeast with and without gustatory intake. The video shows the behavior of a single larva during the entire local search paradigm exposed to yeast (left video), fermented yeast without (middle video) or with feeding possibility (right video). The videos were recorded with two frames per second and playback is sped up by a factor of ten.
